# Whole-Genome Sequencing Reveals the Role of Cis-Regulatory Elements and eQTL/sQTL in the Adaptive Selection of Hubei Indigenous Cattle

**DOI:** 10.3390/ani15091301

**Published:** 2025-04-30

**Authors:** Liangyu Shi, Pu Zhang, Bo Yu, Qing Liu, Chenhui Liu, Wei Lu, Lei Cheng, Hongbo Chen

**Affiliations:** 1 Laboratory of Genetic Breeding, Reproduction and Precision Livestock Farming, School of Animal Science and Nutritional Engineering, Wuhan Polytechnic University, Wuhan 430023, China; liangyu_shi@whpu.edu.cn (L.S.); z15515092327@163.com (P.Z.); wonderfish@whpu.edu.cn (B.Y.); liu3996406@163.com (Q.L.); 2 Institute of Animal Science and Veterinary Medicine, Wuhan Academy of Agricultural Sciences, Wuhan 430208, China; lchhcl890621@sina.com; 3 Hubei Cereals, Oils and Foodstuffs lmport and Export Group Co., Ltd., Wuhan 430015, China; 18971085616@189.cn

**Keywords:** indigenous cattle, adaptive selection, cis-regulatory elements, eQTL, immune, meat quality

## Abstract

Hubei indigenous cattle have adapted to diverse environmental conditions, exhibiting unique genetic traits associated with both economic and adaptive characteristics. Understanding their adaptive selection offers insight into their evolutionary history and genetic enhancements. In this study, we analyzed whole-genome sequencing (WGS) data from five Hubei indigenous cattle breeds to identify selection signals. Selective sweep analysis revealed the candidate genes (*USH2A*, *TMTC2*, *ABCC12*, and *SUGT1*) associated with sensory perception, backfat thickness, reproduction, and immune function. The further integration of cis-regulatory elements (CREs) and expression quantitative trait loci (eQTL) highlighted regulatory variants, influencing adaptive traits. Notably, positively selected genes such as *RPS6KA2*, *CRLS1*, *MGST3*, *GPCPD1*, and *LDLRAP1* were associated with lipid metabolism, meat quality, and reproductive traits, influencing aldehyde volatile organic compounds (VOCs) and fat deposition. These findings highlight the understanding of the genetic basis of adaptation and production traits in Hubei indigenous cattle and provide valuable insights for their conservation and potential breeding strategies.

## Simple Summary

Hubei indigenous cattle exhibit unique adaptations to their local environments. This study investigates the genetic footprints underlying these adaptations by analyzing whole-genome sequences from five local cattle breeds in Hubei. The analysis identified genomic regions under adaptive selection, highlighting genes associated with sensory perception, fat deposition, reproduction, and immune responses. Further examination of cis-regulatory elements revealed their critical role in controlling gene expression related to lipid metabolism, meat quality, and fertility. The findings reveal breed-specific adaptations and support breeding strategies to enhance resilience and meat traits in Hubei indigenous cattle.

## 1. Introduction

Indigenous cattle breeds, evolved under natural and artificial selection, provide valuable genetic insights for understanding the mechanisms of adaptation and evolutionary dynamics [1]. Unlike commercial breeds, which have undergone intensive artificial selection, local breeds retain the signatures of long-term adaptation to diverse ecological conditions. Local breeds often harbor genetic variants offering resilience to environmental stresses such as extreme climates [2,3,4], limited resources [5], or local diseases [6,7]. Therefore, these genetic resources offer us opportunities to investigate the relationships between selection and adaptive genomic regions.

Adaptive selection significantly influences the phenotypic and genetic diversity within and among species [8,9]. Investigating selected genomic regions enables the identification of genes and regulatory elements responsible for specific adaptations, thereby enhancing our understanding of evolutionary processes. Traditionally, studies on adaptive selection have primarily focused on protein-coding genes [10,11,12,13,14]. Emerging evidence highlights the crucial role played by non-coding regions, such as cis-regulatory elements (CREs), epigenomics, in driving phenotypic variation and environmental adaptability [15,16]. CREs enable organisms to adapt to environmental challenges by regulating gene expression, thus serving as critical mediators of adaptive evolution.

Despite the important role played by local breeds for agriculture and biodiversity conservation, their genomic potential remains unexplored. Many local breeds undergo genetic erosion due to crossbreeding [17]. Identifying selection signals in these populations illuminates their evolutionary history, provides insights into genetic diversity, and improves breeding programs. Integrating CREs with genome-wide selection analysis provides a new insight into genotype–phenotype relationships.

In this study, we utilized whole-genome resequencing (WGS) from five geographically diverse local breeds to establish a comprehensive framework for the identification and annotation of adaptive selection signals. Our research primarily focuses on CREs and protein-coding genes, highlighting the important role played by regulatory elements in local breed-specific adaptations. These findings not only enrich our understanding of evolutionary biology but also contribute to developing breeding strategies aimed at enhancing resilience and productivity in livestock populations.

## 2. Materials and Methods

### 2.1. Sample Collection and Genomic Sequencing

Ear tissue samples were collected from a representative cohort of 80 cattle across four distinct breeds originating from Hubei: Dabieshan (*n* = 28), Wuling (*n* = 14), Yiling (*n* = 20), and Yunba (*n* = 18). The data for the Zaobei breed (*n* = 18) have been previously published in our previous research [18]. Each of these samples was obtained from their five core breeding farms. Paired-end sequencing libraries were constructed with an average insert size of 500 bp and a read length of 150 bp for each individual. High-throughput sequencing was carried out on the BGI MGI-T7 platform. To increase the sample size of Hubei indigenous cattle, we downloaded whole-genome sequencing (WGS) data for 10 individuals of Hubei from the National Center for Biotechnology Information (NCBI) database (https://www.ncbi.nlm.nih.gov/ accessed on 20 December 2024). Comparative reference data were obtained from the NCBI database, including 20 Simmental and 18 indicine cattle. Furthermore, two Yak genome data were sourced from the China National Center for Bioinformation (https://www.cncb.ac.cn/ accessed on 20 December 2024). These external samples (Supplementary Table S1) represent commercial taurine and indicine, and outgroup (yak), and were used for population structure, phylogenetic, and selection analyses.

### 2.2. Quality Control, Sequence Alignment, and Variant Calling

Whole-genome resequencing obtained from five cattle breeds was subjected to rigorous quality control using Trimomatic (v0.39) [19], which trimmed adapter sequences and removed low-quality bases. The high-quality reads were then aligned to the cattle reference genome (ARS-UCD1.3), utilizing the BWA-MEM (v0.7.17) [20]. Aligned sequences were sorted and indexed using Samtools (1.16.1) [21], and duplicate reads were identified and purged with Picard (v3.1.0) [22] using the MarkDuplicates.

Variant calling was performed using the GATK (v4.2.6.1) [23]. Individual GVCF files for each sample were generated via HaplotypeCaller [24], and these were subsequently merged into a single VCF file using the CombineGVCFs. SNPs were filtered using VariantFiltration. To minimize false positives, the filtration parameters were set as follows: QD < 2.0, QUAL < 30.0, SOR > 3.0, FS > 60.0, MQ < 40.0, MQRankSum < −12.5, and ReadPosRankSum < −8.0. Non-biallelic SNPs and missing genotype rates > 0.1 were also removed. SNPs phasing was performed using Beagle (v5.4) [25] with default parameters, and SNPs were filtered with DR^2^  <  0.9.

### 2.3. Population Structure and Demographic History Analysis

For PCA and admixture analysis, SNPs with high linkage disequilibrium (LD) were removed using PLINK (v1.90) [26] with the --indep-pairwise 50 10 0. Principal Component Analysis (PCA) was performed with PLINK (v1.90) to assess the genetic structure of the cattle breeds. To further evaluate genetic structure, ADMIXTURE (v1.3) [27] was used, assuming ancestral populations from K = 2 to K = 5. Additionally, a phylogenetic tree was constructed using TreeMix [28] with a maximum likelihood approach to investigate the relationships among the cattle breeds.

The effective population size (*Ne*) for each of the five Hubei breeds, Simmental cattle, and indicine cattle was inferred using SMC + + (v1.15.2) [29]. The mutation rate was 1.25 × 10^−8^ per generation.

Runs of homozygosity (ROH) were analyzed to evaluate inbreeding levels in each individual. ROHs were detected using the PLINK --homozyg, --homozyg-density 100, --homozyg-gap 500, --homozyg-kb 300, --homozyg-window-het 1, --homozyg-window-missing 2, --homozyg-window-snp 50, and --homozyg-window-threshold 0.01 [30]. The genomic inbreeding coefficient (*F*_ROH_) = ∑L_ROH_/L_GENOME_.

Linkage disequilibrium (LD) in each population was estimated by calculating the squared correlation coefficients (*r*^2^) between SNP pairs using PopLDdecay (v3.43) [31] with default settings. LD patterns in the genomic regions of interest were visualized using LDBlockShow (v1.40) [32].

### 2.4. Theta Pi

We used VCFtools [33] to estimate nucleotide diversity for each breed, employing window sizes of 50 kb with 20 kb step while keeping other parameters at default settings. The π-ratio [34] was calculated as the ratio of nucleotide diversity between Simmental and Hubei indigenous cattle (π_Simmental_/π_Hu_) and between indicine cattle and Hubei indigenous cattle (π_Indicine_/π_Hu_). The identified key genes were analyzed and illustrated using HTIRDB (https://yanglab.hzau.edu.cn/HTIRDB#/expression/single_all_species, accessed on 24 February 2025) [35].

### 2.5. Identification of Adaptive Selections

Adaptive selection in cattle was analyzed using six statistical methods, incorporating both recent and long-term selection events. Adaptive regions were defined as the union of all significant loci or windows identified by these methods.

To detect recent hard sweeps, we utilized iHS [36], nSL [37], and CLR [38]. These methods were calculated through selscan (v2.0.0) [39,40] and normalized to identify regions exhibiting significant deviations from neutrality. For soft sweeps, the iHH12 [41] was computed. To ensure that the focus was on regions under positive selection, we selected loci with a minor allele frequency (MAF) greater than 0.01 for statistical testing. For |iHS|, nSL, and iHH12, *p*-values were computed and thresholds were set at *p* < 0.005 (Z-test) to identify candidate signatures of selection. CLR was performed across 50 kb windows with 20 kb steps, with significant selection signals also determined by *p*-values less than 0.005.

For identifying signals of long-term balance selection, we focused on genomic foci with MAF greater than 0.15 [42]. Beta statistics were calculated for these regions, and coupled with Tajima’s D [43] greater than 2, were used to identify significant balance selection signals under the threshold of *p* < 0.005 (right-tailed interval). In our analysis, the ancestral allele was defined as ARS-UCD1.3 [42].

Additionally, the pairwise fixation index (*F*_ST_) was calculated using VCFtools [33], with a sliding window of 50 kb with a 20 kb step size to evaluate genetic differentiation between the breeds, while keeping other parameters at default settings.

### 2.6. Cis-Regulatory Element Annotations and Function QTL

The cis-regulatory element (CRE), which includes various tissues and regulatory components, was obtained from Kern et al. [44]. The tissues comprised adipose, cerebellum, cortex, hypothalamus, liver, lung, muscle, and spleen.

Expression quantitative trait loci (eQTL) and splicing quantitative trait loci (sQTL) were sourced from the FarmGTEX database [45], which includes 37 tissues: adipose, blood, colon, conceptus, embryo, hypothalamus, ileum, jejunum, kidney, leukocyte, liver, lung, lymph node, macrophage, mammary (left and general), milk cells, monocytes, various muscle types (Muscle_Cesar_et_al, Muscle_cross, Muscle_indicus, Muscle_taurus, general muscle), nasal pharynx, oocyte, ovary, oviduct, PBMC, pharyngeal tonsil, pituitary, rumen, salivary gland, skin fibroblast, small intestine, spleen, testis, and uterus.

### 2.7. Definition of Adaptive Regions and Identification of Candidate Adaptive CREs

Genomic regions under adaptive selection were defined as “adaptive regions”. To further delineate potential adaptive variants, genomic signatures were integrated with functional genomic data. These regions may contribute to fitness-related adaptations due to environmental pressures or genetic linkage with causal adaptive variants. For window-based selection analyses, adaptive regions were identified as statistically significant selection signals. For locus-based selection analyses, adaptive regions were defined as the 1 kb upstream and downstream regions of the loci where selection was significant.

CREs located within adaptive regions and exhibiting significant selection signals were classified as candidate adaptive CREs [42]. iSAFE analysis was performed within adaptive CREs, and the top 5% of iSAFE signals were considered adaptive regions influencing these CREs. CRE-regulated genes identified in the five Hubei indigenous cattle breeds were visualized using UpSet [46].

### 2.8. Functional Enrichment Analysis of Adaptive CREs

To investigate the functional relevance of adaptive regions contributing to the regulation of CREs, Gene Ontology (GO) and KEGG pathway enrichment analyses were performed using WebGestalt [47], with a significance threshold of FDR < 0.05.

## 3. Results

### 3.1. Sequencing and Variation Calling

**Figure 1:**
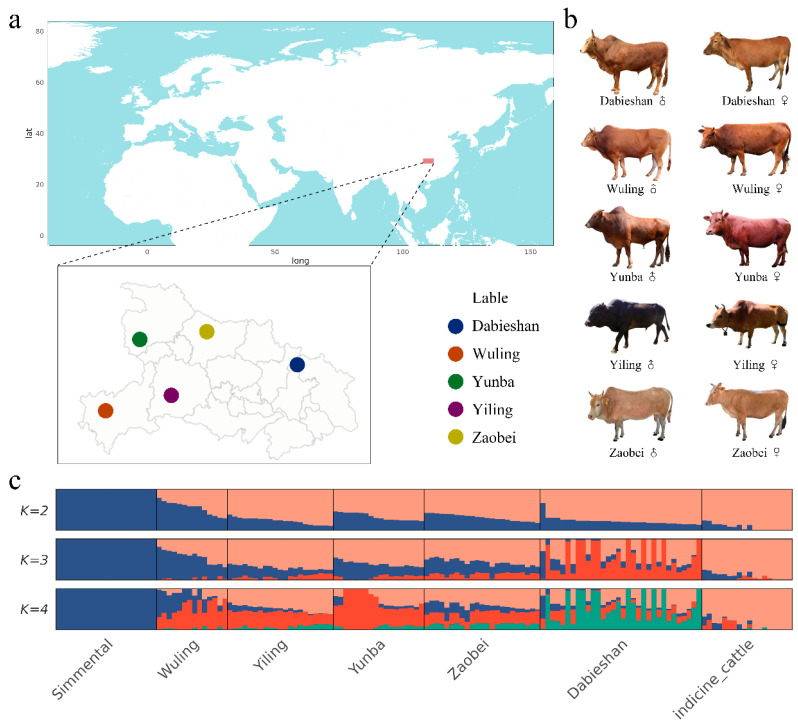
Geographical distribution and admixture of the cattle breeds. (**a**) Sampling location for the five Hubei indigenous cattle breeds. (**b**) Breed photographs of male and female animals of the five Hubei indigenous cattle breeds. (**c**) ADMIXTURE of all 146 cattle using LD-pruned SNPs for K from 2 to 4.

A total of 3.01 Tb of clean sequencing data was generated from the individual genomes of the 98 individuals from five cattle breeds (Figure 1a,b; Supplementary Table S2). On average, each sample generated 50,247,006 reads, with 50,113,037 reads (99.73%) successfully mapped to the reference genome. The mean sequencing depth across all individuals ranged from 17.8×~28.7×, with an average genomic coverage of 0.97. SNP annotation revealed that the majority of SNPs were located in non-coding areas, with 64.36% in intergenic and 31.76% in intronic regions, indicating their roles in regulatory functions.

### 3.2. Population Structure and Genetic Diversity

Our genomic analyses covered diverse cattle breeds, including Dabieshan, Yunba, Yiling, Zaobei, Wuling, indicine cattle, and Simmental to assess population structure and genetic diversity. Principal Component Analysis (PCA) distinguished Hubei indigenous cattle breeds from indicine cattle and Simmental (Supplementary Figure S1). ADMIXTURE revealed four main ancestral components at K = 4, with the lowest cross-validation error of 0.307 (Figure 1c; Supplementary Figure S2). A similar trend was observed in the maximum likelihood (ML) phylogenetic tree (Supplementary Figure S3).

**Figure 2:**
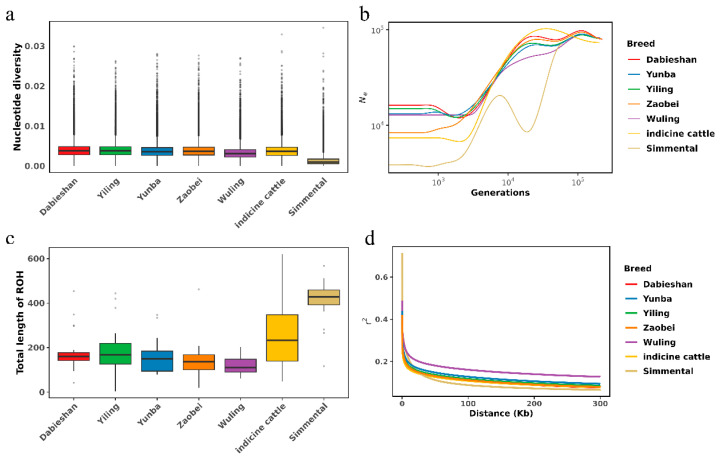
Population genetic diversity and linkage disequilibrium patterns among seven cattle breeds/populations, including Dabieshan, Yunba, Yiling, Zaobei, Wuling, indicine cattle, and Simmental. (**a**) Nucleotide diversity (π). (**b**) Effective population size (Ne). (**c**) Total length of runs of homozygosity (ROH). (**d**) Linkage disequilibrium decay (*r*^2^) across genomic distances (Kb) for each breed.

Nucleotide diversity was generally low, with indicine cattle showing slightly higher diversity, whereas Simmental had lower diversity (Figure 2a). Genetic distances estimated via the pairwise fixation index (*F*_ST_) ranged from 0.01 to 0.04 among five Hubei indigenous cattle breeds (Supplementary Figure S4). Wuling had the lowest *F*_ROH_, while Simmental had the highest, suggesting more intensive recent inbreeding or selection in Simmental (Figure 2c).

The effective population size (*N*_e_) estimates demonstrated a sharp delay over the last ~10,000 generations (Figure 2b). Wuling cattle exhibited the slowest LD decay, while Zaobei decayed below *r*^2^ = 0.2 rapidly compared to other breeds (Figure 2d).

### 3.3. Genome-Wide Selective Sweep

Genome-wide selective sweep distinguished Hubei indigenous cattle breeds from indicine cattle and Simmental. According to the θ_π_ ratio, significant genomic regions were identified based on the top 5% threshold. π_Hu_ was reduced in Hubei cattle, while Simmental and indicine cattle maintained higher levels of genetic diversity. This contrast led to high θπ ratios and extended regions of nearly fixed haplotypes in Hubei cattle, which was consistent with a recent hard selective sweep. Genes within these selected regions showed distinct θ_π_ and haplotype patterns, compared to Simmental and indicine cattle, particularly Simmental. These findings supported the hypothesis that the adaptive evolution of Hubei cattle has been driven by recent and breed-specific positive selection rather than by the retention of ancestral variants commonly shared with commercial breeds.

**Figure 3:**
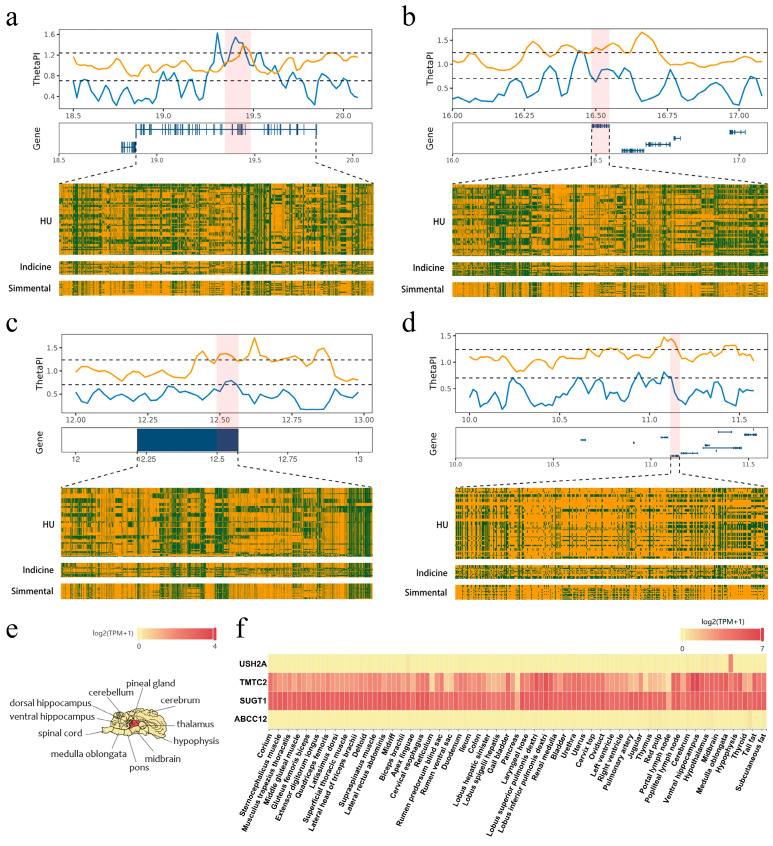
Genomic differentiation and selective sweep analysis. (**a**–**d**) Nucleotide diversity (θ_π_) values plotted using a sliding window approach, comparing π_Simmental_/π_Hu_ (blue) and π_Indicine_/π_Hu_ (yellow). Candidate selective sweep regions are highlighted in light red. Haplotype structures of SNPs in the selected regions, where yellow represents reference alleles and green represents alternative alleles. (**e**) Brain tissue-specific expression patterns of *USH2A*. (**f**) Heatmap of tissue-specific gene expression for key candidate genes (*USH2A*, *TMTC2*, *SUGT1*, *ABCC12*) across various tissues.

Gene annotation within the selected regions revealed 37 genes shared between Simmental and Hubei indigenous cattle (π_Simmental_/π_Hu_), as well as in indicine cattle and Hubei indigenous cattle (π_Indicine_/π_Hu_). *USH2A* gene encodes a protein called usherin, which is integral to the development and maintenance of the inner ear and retina (Figure 3a). Usherin is a component of basement membranes that separate and support cells in various tissues. *USH2A* is highly expressed in the pineal gland and medulla oblongata (Figure 3e,f). The *ABCC12* gene encodes the protein Multidrug Resistance-Associated Protein 9 (MRP9), which belongs to the ATP-binding cassette (ABC) transporter superfamily (Figure 3b). The absence or dysfunction of MRP9 led to reduced fertility and abnormal sperm development. The *TMTC2* gene encodes a protein located in the endoplasmic reticulum (ER) membrane (Figure 3c). This protein contains multiple tetratricopeptide repeats, which facilitate protein–protein interactions. *SUGT1* encodes a conserved nuclear protein involved in kinetochore function and is essential for cell cycle transitions (Figure 3d).

### 3.4. The Genomic Region Under Adaptive Selection in the Hubei Indigenous Cattle Breeds

**Figure 4:**
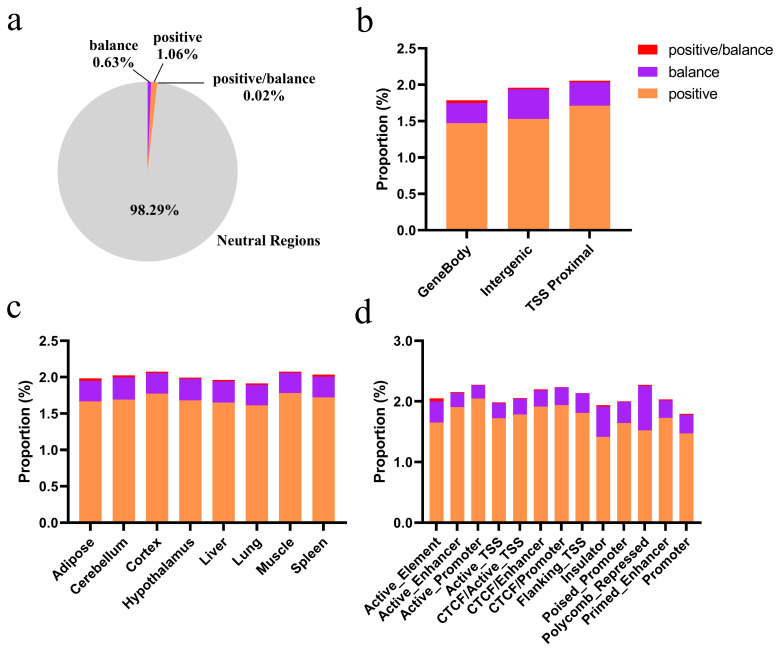
Distribution of adaptive selection signals across genomic regions and CREs in Yiling cattle. (**a**) Proportion of genomic regions under positive selection, balance selection, and both selection types. (**b**) Distribution of selection signals across gene body, intergenic, and TSS-proximal. (**c**) Tissue-specific distribution of adaptive selection signals. (**d**) Selection signal coverage across different CRE types. The Y-axis represents the proportion calculated as the total length of adaptive selection regions divided by the total length of each functional category.

To identify genomic regions significantly under adaptive selection in each of the Hubei indigenous breeds, we applied six methods: iHS (Supplementary Figure S5), nSL (Supplementary Figure S6), CLR (Supplementary Figure S7), ihh12 (Supplementary Figure S8), Beta (Supplementary Figure S9), and Tajima’s D (Supplementary Figure S10). The adaptive selection regions in the five local breeds ranged from 35.20 to 71.72 Mb. The majority of genomic regions (>97%) were classified as neutral (Figure 4a; Supplementary Figures S11a–S14a). Positive selection signals ranged from 0.58% to 2.05%, with the highest observed in Yunba cattle (2.05%), and the lowest in Zaobei (0.58%). Balance selection signals varied between 0.42% and 1.02%, wherein Dabieshan cattle showed the highest (1.02%) and Zaobei the lowest (0.63%). The proportion of regions under both positive and balance selection was consistently low (~0.01% to 0.04%).

We further analyzed adaptive selection signals in CREs across various tissues. Adaptive selection signals in the gene body showed the lowest proportion across all breeds, and the proportion of positive selection signals was consistently higher than the balance selection signals in each type of CRE (Figure 4b; Supplementary Figures S11b–S14b). Among the breeds, Yunba cattle exhibited the highest proportion of selection signals in all three CRE types, particularly in TSS-proximal, where the proportion is 3.66%. In contrast, Zaobei cattle had the lowest selection signal proportions, with TSS-proximal accounting for ~1.04%. The proportions of adaptive selection signals were similar across CRE-associated tissues within each breed (Figure 4c; Supplementary Figures S11c–S14c). Dabieshan and Yunba cattle showed slightly higher proportions in the spleen, whereas Wuling cattle exhibited slightly higher adaptive selection signal coverage in the muscle. Among various CREs, active promoters and enhancers exhibited the largest selection signal coverage, and CTCF-bound enhancers and polycomb-repressed also exhibited high selection signal coverage (Figure 4d; Supplementary Figures S11d–S14d). CTCF enhancers (3.73%) showed the largest selection in Yunba cattle, while polycomb-repressed had the lowest selection in Zaobei cattle (1.57%).

**Figure 5:**
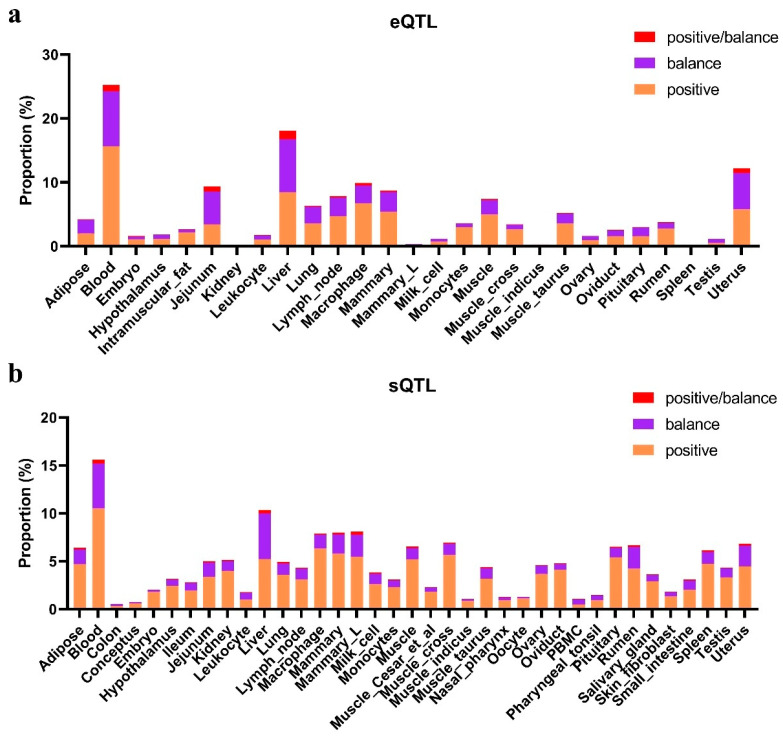
Proportion of expression quantitative trait loci (eQTL) and splicing quantitative trait loci (sQTL) across different tissues in Yiling cattle. (**a**) Distribution of eQTL proportions in various tissues. (**b**) Distribution of sQTL proportions in the same tissues. The bars represent the proportion of positive (orange), balance (purple), and positive/balance (red) QTL effects in each tissue. The Y-axis represents the proportion calculated as the total length of adaptive selection regions divided by the total length of each functional category.

The distribution of expression quantitative trait loci (eQTL) and splicing quantitative trait loci (sQTL) mapping to adaptive selection signals was highest in the blood across all breeds, followed by the liver (Figure 5; Supplementary Figures S15–S18). The uterus and muscle also showed relatively high proportions of eQTL. In the muscle, the Dabieshan breed had the highest number of related genes (2211 genes), followed by Yiling, while the Wuling breed had the lowest (1999 genes). In the uterus, the highest overlap was observed in the Dabieshan breed, followed by Zaobei, whereas the Wuling breed had the fewest related genes. To further investigate the functional relevance of the genes identified in the muscle and uterus, we performed GO/KEGG enrichment analysis (Supplementary Table S3). In Zaobei cattle, two significantly enriched pathways were detected in the muscle, fatty acid metabolism (bta01212) and fructose and mannose metabolism (bta00051), both of which are closely related to lipid metabolism. Similarly, multiple candidate genes associated with volatile organic compounds (VOCs) have been identified in Chinese Jingxing Yellow (JXY) chickens, many of which are involved in fatty acid metabolism and phospholipid pathways [48].

### 3.5. Candidate Positively Selected CREs in the Hubei Indigenous Cattle Breeds

**Figure 6:**
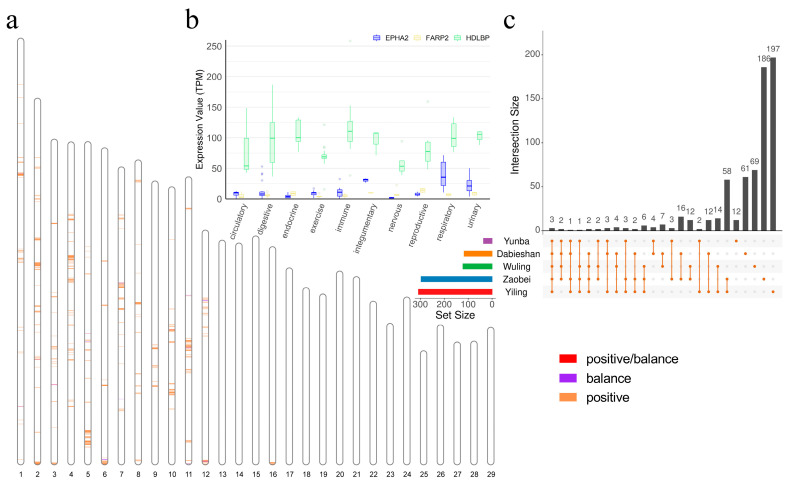
Distribution of candidate region in positively selected CREs and associated genes. (**a**) Distribution of candidate regions in positively selected CREs in Yiling cattle. (**b**) Genes associated with positively selected CREs across Hubei indigenous cattle breeds. (**c**) Gene expression across different physiological systems for *EPHA2*, *HDLBP*, and *FARP2*.

The distribution of candidate positively selected CREs showed a higher density in Yiling cattle, particularly on chromosomes 1 to 12, while Yunba cattle had fewer detected candidate CREs (Figure 6a; Supplementary Figure S15). The genes *EPHA2*, *HDLBP*, and *FARP2* were identified within positively selected CRE regions in all breeds (Figure 6b). Of these genes, *HDLBP* showed the highest overall expression in all systems, *EPHA2* exhibited the highest levels observed in the respiratory systems, and *FARP2* had consistently low expression across most systems, with slightly higher levels in the reproductive systems (Figure 6c). Additionally, *CTDSP1* was not identified in Dabieshan and Zaobei, *SHBG* was absent in Wuling, and *HSPA2* was not detected in Zaobei.

Pathway analysis revealed significant enrichment in Yiling cattle of genes regulated by positively selected CREs such as serine/threonine protein kinase complex, carboxy–terminal domain protein kinase complex, and protein kinase complex (FDR < 0.05). Additionally, the MAPK signaling pathway and pentose and glucuronate interconversions (FDR = 0.10) were also detected, though they were not statistically significant. In other cattle breeds, GO and KEGG annotations of candidate positively selected CRE-regulated genes were not significant (Supplementary Table S4).

## 4. Discussion

In this study, we conducted whole-genome sequencing of 98 individuals from five Hubei indigenous cattle breeds to assess their genetic diversity, population structure, and potential adaptive regions. Our analysis revealed low genetic differentiation among these breeds, which is attributed to shared ancestry, gene flow between populations, or similar selective pressures across Hubei indigenous cattle breeds.

The contrasting nucleotide diversity (π) and inbreeding coefficients (*F*_ROH_) among breeds reflect their different breeding practices, gene flow, and selection pressures. Commercial Simmental cattle exhibited the lowest π and highest *F*_ROH_, which was consistent with intensive artificial selection targeting production traits. The five Hubei indigenous breeds showed generally low nucleotide diversity, but their *F*_ROH_ varied by breed. Wuling cattle had the lowest *F*_ROH_, likely due to historically large local population sizes and ongoing gene flow among neighboring herds. Yiling cattle displayed the highest *F*_ROH_, suggesting stronger selection pressures, which can be observed in some commercial breeds.

Hubei indigenous cattle have undergone long-term adaptation to humid, pathogen-rich environments. This evolutionary history has resulted in heterogeneous haplotype structures at key loci, contrasting with the haplotypes observed in commercial breeds. *USH2A* is highly expressed and plays important functional roles in the human retina and inner ear [49,50]. It has also been implicated in circadian rhythm regulation [51,52] and linked to hair color variation [53], which may influence thermal regulation and camouflage [54]. Although *USH2A* has not yet been reported in cattle, *USH2A* potentially contributes to reproductive performance in Hubei indigenous cattle. *TMTC* is reported to regulate calcium homeostasis [55], which is essential for skeletal and muscular development in mice [56]. This conserved function may explain its associations with body conformation traits in Holstein bulls [57] and backfat thickness in Simmental cattle [58]. According to the HTIRDB database [35], *MRP9* is highly expressed in the testis and sperm mitochondria in cattle. Together with *MRP5*, it contributes to sperm function and fertility, and their dysfunction leads to reduced reproductive capacity [59]. *SUGT1* is a positive regulator of both MHC class I and class II surface expression, influencing the stability and expression of key transcription factors involved in MHC gene regulation [60]. This function may contribute to disease resistance in Hubei indigenous cattle.

Although the same window size and significance threshold were applied across all autosomes, we found that most positively selected cis-regulatory elements (CREs) were located on chromosomes 1 to 12. These enrichments are unlikely due to statistical bias. These characteristics likely made them stronger targets of selection in Hubei indigenous cattle. The genes *EPHA2*, *HDLBP*, and *FARP2* were identified within positively selected CRE regions in all five Hubei indigenous cattle breeds. *EPHA2* was identified as a target gene of miR-26b, showing an inverse expression pattern in Yanbian cattle, suggesting a role in pituitary hormone secretion and development [61]. In mice [62] and yaks [63], *EPHA2* is involved in cardiovascular development through epigenetic regulation and in hypoxia adaptation via pulmonary artery remodeling, respectively. These findings suggest that *EPHA2* may also contribute to environmental adaptation in Hubei indigenous cattle. *HDLBP*, which encodes the RNA-binding protein vigilin, plays a multifaceted role in cellular processes such as translation, chromosome segregation, cholesterol transport, and carcinogenesis [64]. Although studies on this gene in cattle are limited, evidence from human and mouse models has shown that HDLBP regulates VLDL secretion by controlling ApoB mRNA translation [65], indicating a potentially conserved role in bovine lipid metabolism. *FARP*, a guanine nucleotide exchange factor (GEF), activates RAC1, a key regulator of the actin cytoskeleton, and is involved in cell migration, osteoclast differentiation, and integrin signaling. In duck ovarian tissue, miRNA novel_221 participates in a regulatory network, including the *FARP2* gene [66].

Among the five Hubei indigenous cattle breeds, only Yiling cattle have been investigated for meat quality, thus providing an effective model to study the impact of CRE-related genes on this characteristic. Yiling cattle, native to Yichang, Hubei Province, are known for their high-marbling beef [67]. The volatile organic compounds (VOCs) present in Yiling beef indicate a complex and distinctive aroma, with higher levels of alcohols, aldehydes, and high-molecular-weight hydrocarbons compared to other breeds [68]. The formation of aldehydes, which are key VOCs contributing to meat flavor, is closely linked to lipid metabolism. Among the genes identified in the adaptive selection regions of Yiling cattle, *RPS6KA2*, *CRLS1*, *MGST3*, *GPCPD1*, and *LDLRAP1* were linked to aldehyde production, suggesting their potential role in flavor [48]. Additionally, *PRKAG3* and *CTDSP1*, known to regulate meat pH and color in pigs [69,70], may have similar functions with Yiling cattle. *ALDH2* suggests its role in aldehyde metabolism by breaking down reactive aldehydes and its involvement in AMPK signaling [71], which may influence muscle energy regulation, lipid metabolism, and meat quality. These genes regulate key reproductive processes in Yiling cattle. Beyond their exceptional meat quality, the reproductive traits of Yiling cattle are also undergoing adaptive selection. *GDF9* supports ovarian follicle development [72,73,74] and oocyte maturation [75]. *INSL6* is essential for spermatogenesis [76] and male fertility [77]. *SHBG* encodes a steroid-binding protein, and the encoded protein transports androgens and estrogens [78]. Transcriptional control is mediated by *PRDM1*, which plays a role in germ cell development [79,80]. *HSPA2* ensures proper protein folding during sperm maturation [81]. Pathway enrichment analysis for muscle eQTL-related genes in Zaobei cattle highlighted significant associations with fatty acid metabolism (bta01212) and fructose and mannose metabolism (bta00051), suggesting conserved roles in lipid metabolism and muscle energy regulation. Given the shared regulatory elements and selection signals, the genetic mechanisms identified in Yiling cattle may also apply to Dabieshan, Wuling, Yunba, and Zaobei cattle. These overlaps suggest common adaptive pressures despite environmental differences. While breed-specific phenotypic data are needed for validation, current evidence supports the broader relevance of Yiling-derived regulatory and splicing variants across Hubei indigenous cattle.

The GO/KEGG functional enrichment of candidate positively selected CRE-regulated genes in Yiling cattle also suggests genetic mechanisms influencing meat quality and VOC characteristics. The enrichment of the serine/threonine protein kinase complex, carboxy–terminal domain protein kinase complex, and protein kinase complex may regulate muscle metabolism and fat deposition, affecting marbling and texture. The MAPK signaling pathway and pentose and glucuronate interconversions suggest a potential role in VOC composition, which may contribute to the distinct aroma and flavor of Yiling cattle. These findings indicate that adaptive selection in Yiling cattle may have shaped biological processes related to meat quality and sensory traits.

We acknowledge several limitations in this study. The sample size (n = 98) may limit the statistical power to detect rare variants and weak regulatory effects. A larger sample size is needed to confirm low-frequency signals. The regulatory datasets (cis-regulatory elements, expression QTLs, and splicing QTLs) used are mainly derived from commercial taurine breeds, which may not represent regulatory variants in Hubei indigenous cattle. Due to the lack of breed- and tissue-specific regulatory maps for these populations, the annotation of selection signals using the FarmGTEx multi-tissue dataset is preliminary. Although core regulatory features and eQTLs are often conserved across breeds, breed-specific regulatory variants and splicing events may not be captured. Generating high-resolution regulatory datasets in indigenous breeds using methods such as ATAC-seq, ChIP-seq, and transcriptomic QTL mapping will help verify the regulatory mechanisms identified in this study.

## 5. Conclusions

This study provides a detailed analysis of adaptive selection in five indigenous cattle breeds from Hubei, using whole-genome sequencing and functional genomic annotations. Selective sweep analyses revealed candidate genes involved in reproduction, immunity, and backfat thickness. The integration of cis-regulatory elements (CREs) and expression quantitative trait loci (eQTL) data further highlighted the role of regulatory variants in traits related to muscle metabolism and lipid storage, which are crucial for meat quality. Notably, positively selected genes in Hubei indigenous cattle were associated with marbling and volatile organic compounds (VOCs) that influence meat flavor. These findings offer novel insights into the adaptive evolution and economically important traits of Hubei indigenous cattle, laying a valuable foundation for their conservation and breeding programs.

## Data Availability

The data presented in this study are available on request from the corresponding author.

## Supplementary Materials

The following supporting information can be downloaded at: https://www.mdpi.com/article/10.3390/ani15091301/s1. Table S1. Samples from database; Table S2. Samples and SNPs information; Table S3. Enrichment Analysis of Gene Pathways Regulated by eQTL in Muscle and Uterus Tissues of in Hubei Indigenous Cattle Breeds; Table S4. Gene Ontology (GO) Biological Processes of Cis-Regulatory Elements (CREs) Under Positive Selection in Hubei Indigenous Cattle Breeds; Figure S1. Principal component analysis (PCA) of all 146 cattle using LD-pruned SNPs; Figure S2. Cross-validation error rates from ADMIXTURE analyses for assumed ancestral populations (K) ranging from 2 to 5; Figure S3. TreeMix relationships among 8 cattle breeds/populations; Figure S4. Mean pairwise FST values between Hubei indigenous cattle breeds; Figure S5. Manhattan plots of iHS statistics in Hubei indigenous cattle breeds; Figure S6. Manhattan plots of nsl statistics in Hubei indigenous cattle breeds; Figure S7. Manhattan plots of CLR statistics in Hubei indigenous cattle breeds; Figure S8. Manhattan plots of ihh12 statistics in Hubei indigenous cattle breeds; Figure S9. Manhattan plots of Beta statistics in Hubei indigenous cattle breeds; Figure S10. Manhattan plots of Tajima’s D statistics in Hubei indigenous cattle breeds; Figure S11. Distribution of selection signals across genomic regions and cis-regulatory elements (CREs) in Dabieshan cattle; Figure S12. Distribution of selection signals across genomic regions and cis-regulatory elements (CREs) in Wuling cattle; Figure S13. Distribution of selection signals across genomic regions and cis-regulatory elements (CREs) in Yunba cattle; Figure S14. Distribution of selection signals across genomic regions and cis-regulatory elements (CREs) in Zaobei cattle; Figure S15. Proportion of expression quantitative trait loci (eQTL) and splicing quantitative trait loci (sQTL) across different tissues in Dabieshan cattle Figure S16. Proportion of expression quantitative trait loci (eQTL) and splicing quantitative trait loci (sQTL) across different tissues in Wuling cattle; Figure S17. Proportion of expression quantitative trait loci (eQTL) and splicing quantitative trait loci (sQTL) across different tissues in Yunba cattle; Figure S18. Proportion of expression quantitative trait loci (eQTL) and splicing quantitative trait loci (sQTL) across different tissues in Zaobei cattle; Figure S19. Distribution of Candidate region in Positively Selected CREs in Dabieshan cattle; Figure S20. Distribution of Candidate region in Positively Selected CREs in Wuling cattle; Figure S21. Distribution of Candidate region in Positively Selected CREs in Yunba cattle; Figure S22. Distribution of Candidate region in Positively Selected CREs in Zaobei cattle.
