# Supplementary material for: Whole-Genome Sequencing Reveals the Role of Cis-Regulatory Elements and eQTL/sQTL in the Adaptive Selection of Hubei Indigenous Cattle
Source: Animals (Basel). 2025 Apr 30;15(9):1301. doi: 10.3390/ani15091301 (PMC12071175; doi:10.3390/ani15091301)
Supplement: Supplementary file 1 [file animals-15-01301-s001.zip › Supplementary Figures.pdf]

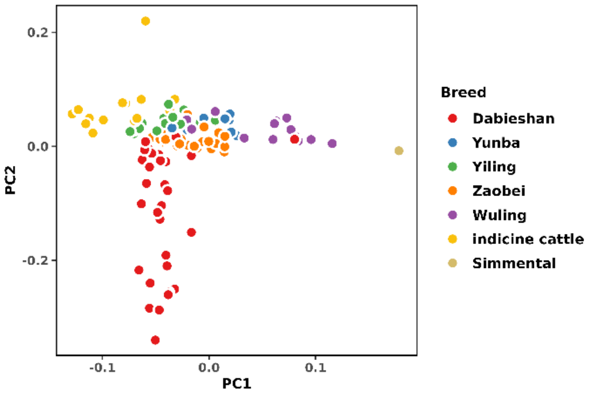

Supplementary Figure S1 Principal component analysis (PCA) of all 146 cattle using LD-pruned SNPs.

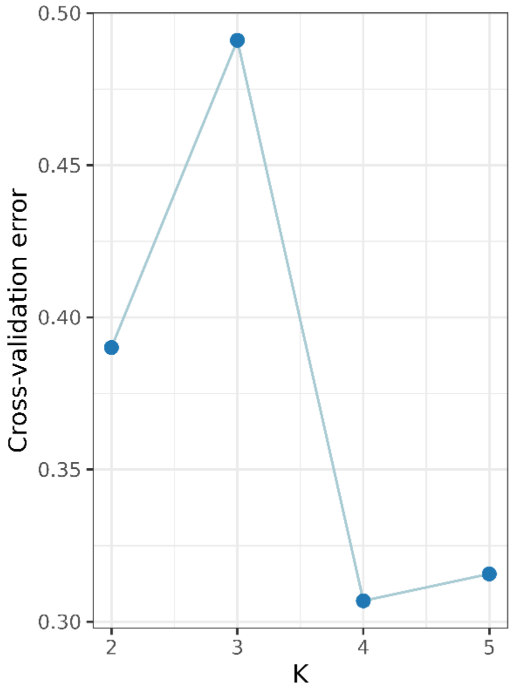

Supplementary Figure S2 Cross-validation error rates from ADMIXTURE analyses for assumed ancestral populations (K) ranging from 2 to 5.

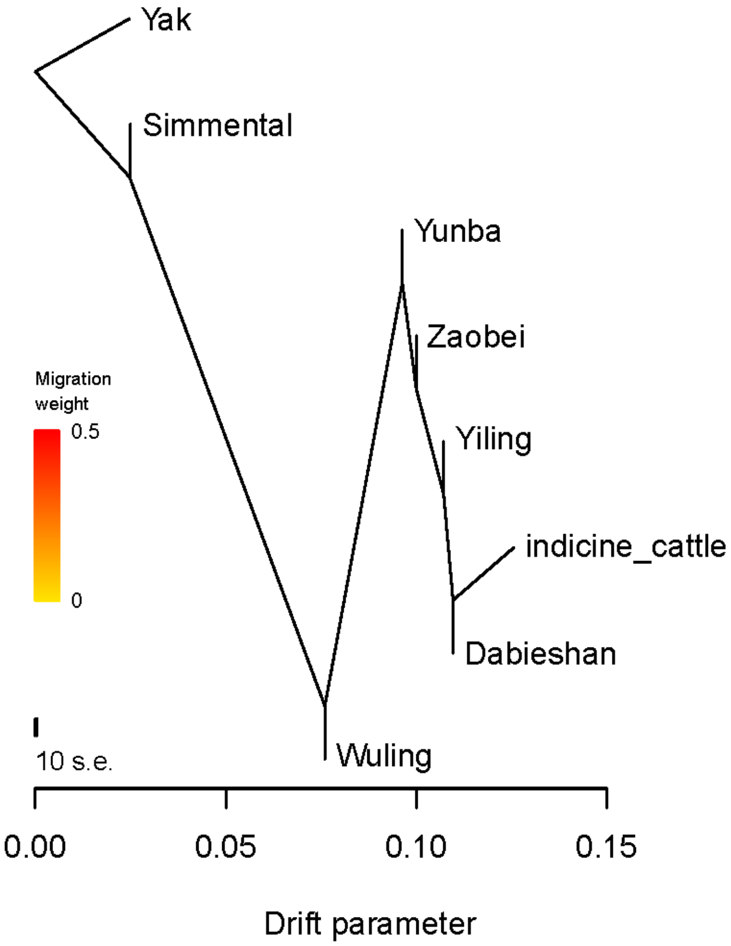

Supplementary Figure S3 TreeMix relationships among 8 cattle breeds/populations.

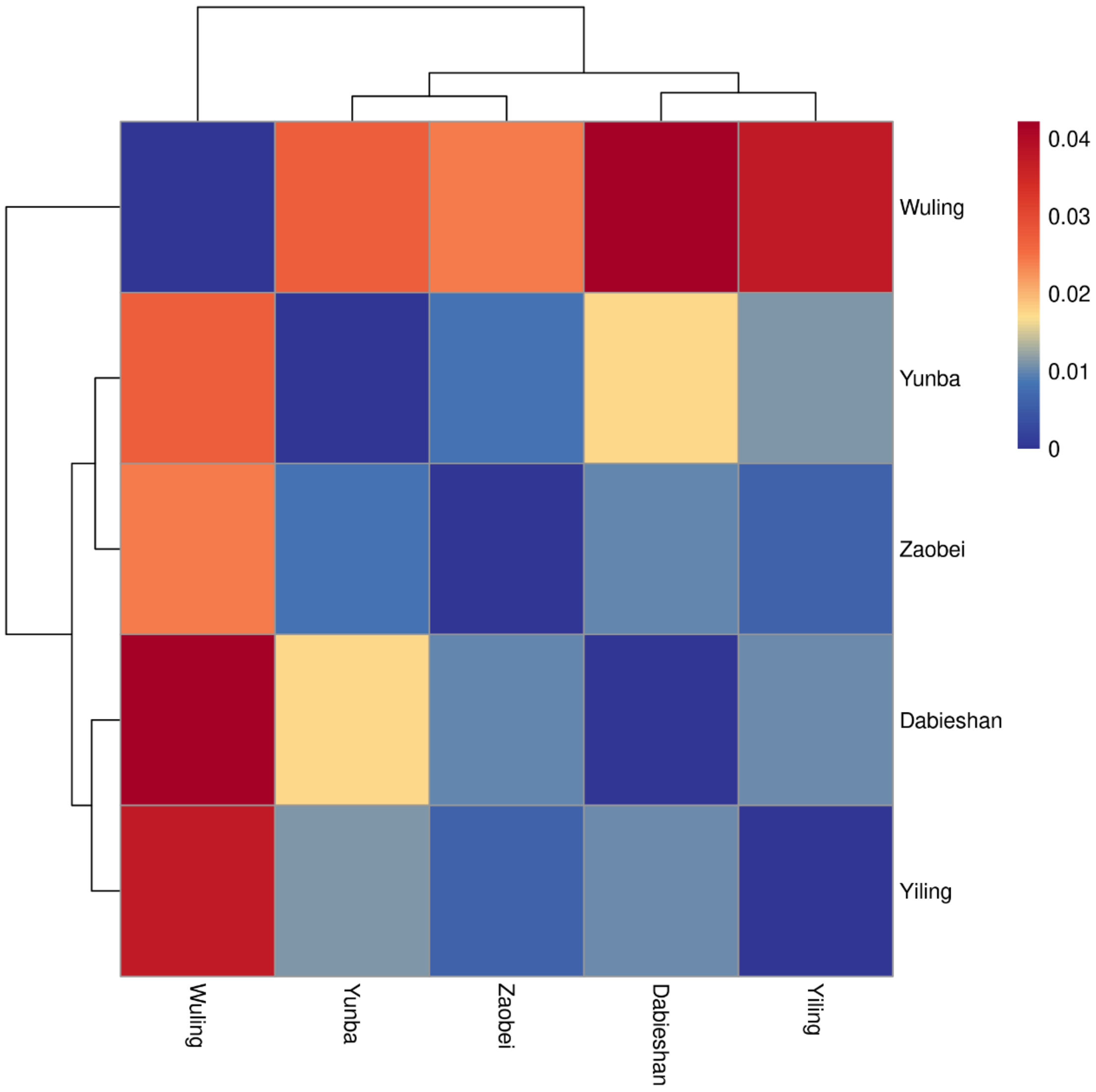

Supplementary Figure S4 Mean pairwise FST values between Hubei indigenous cattle breeds.

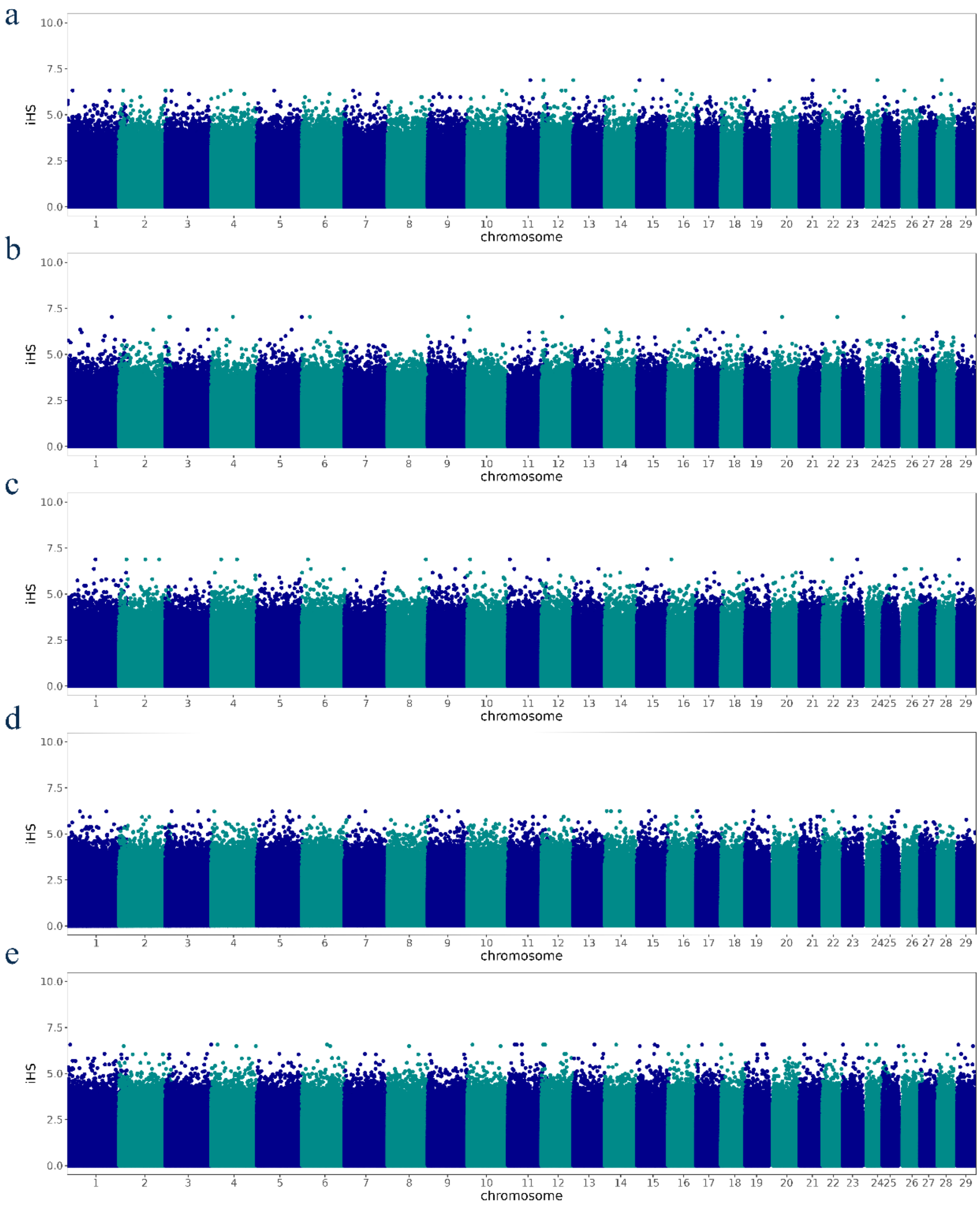

Supplementary Figure S5 Manhattan plots of iHS statistics in Hubei indigenous cattle breeds.

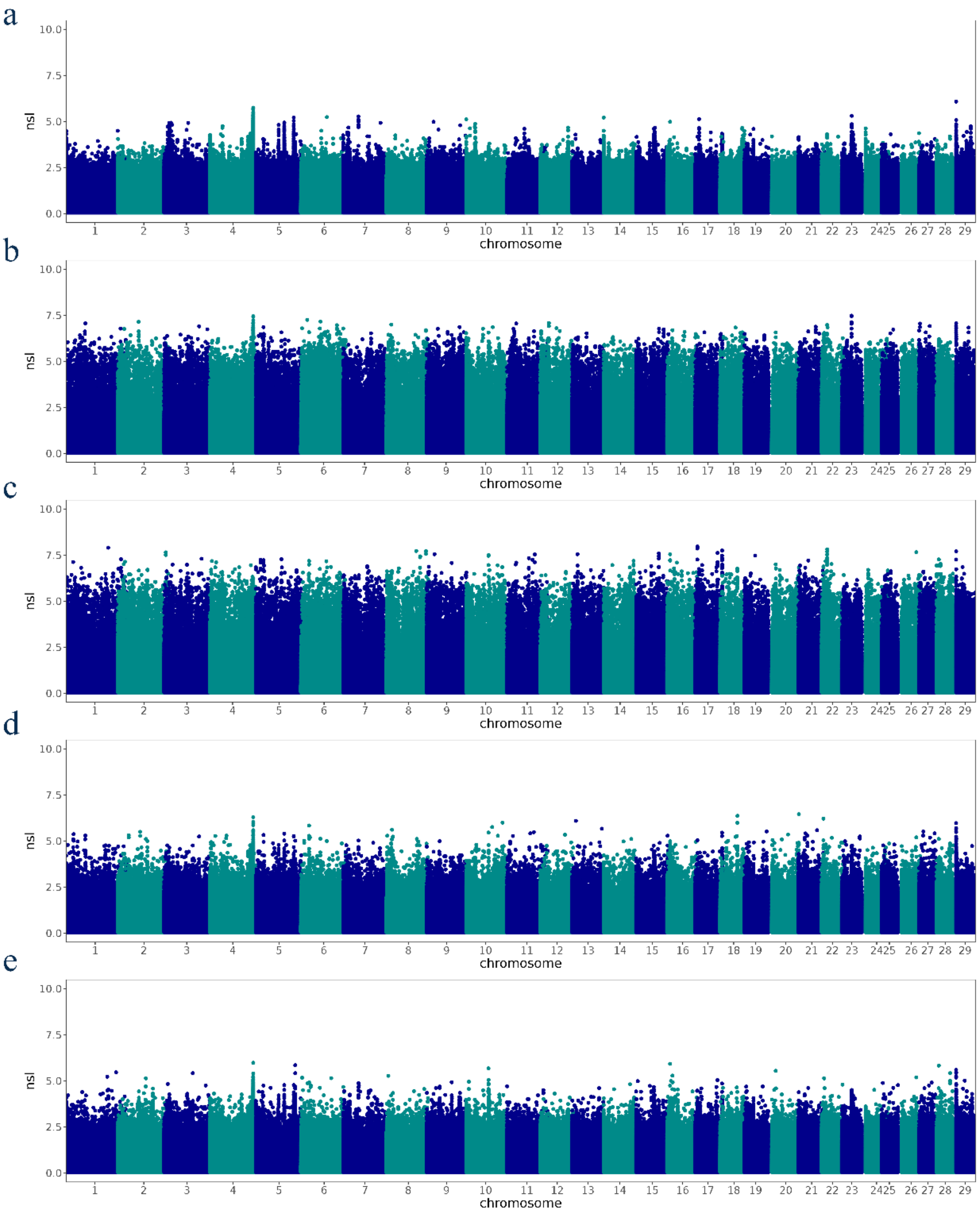

Supplementary Figure S6 Manhattan plots of nsl statistics in Hubei indigenous cattle breeds.

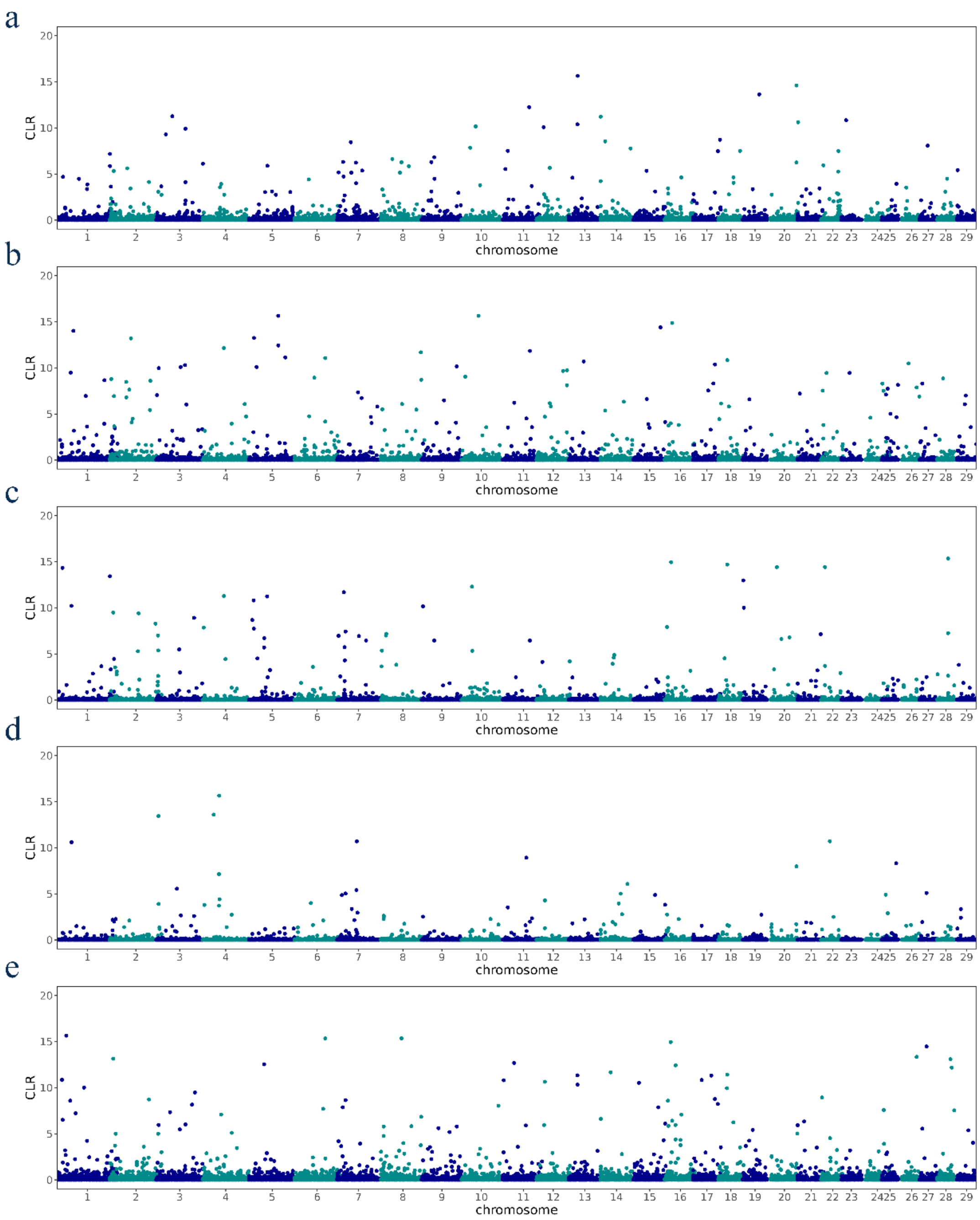

Supplementary Figure S7 Manhattan plots of CLR statistics in Hubei indigenous cattle breeds.

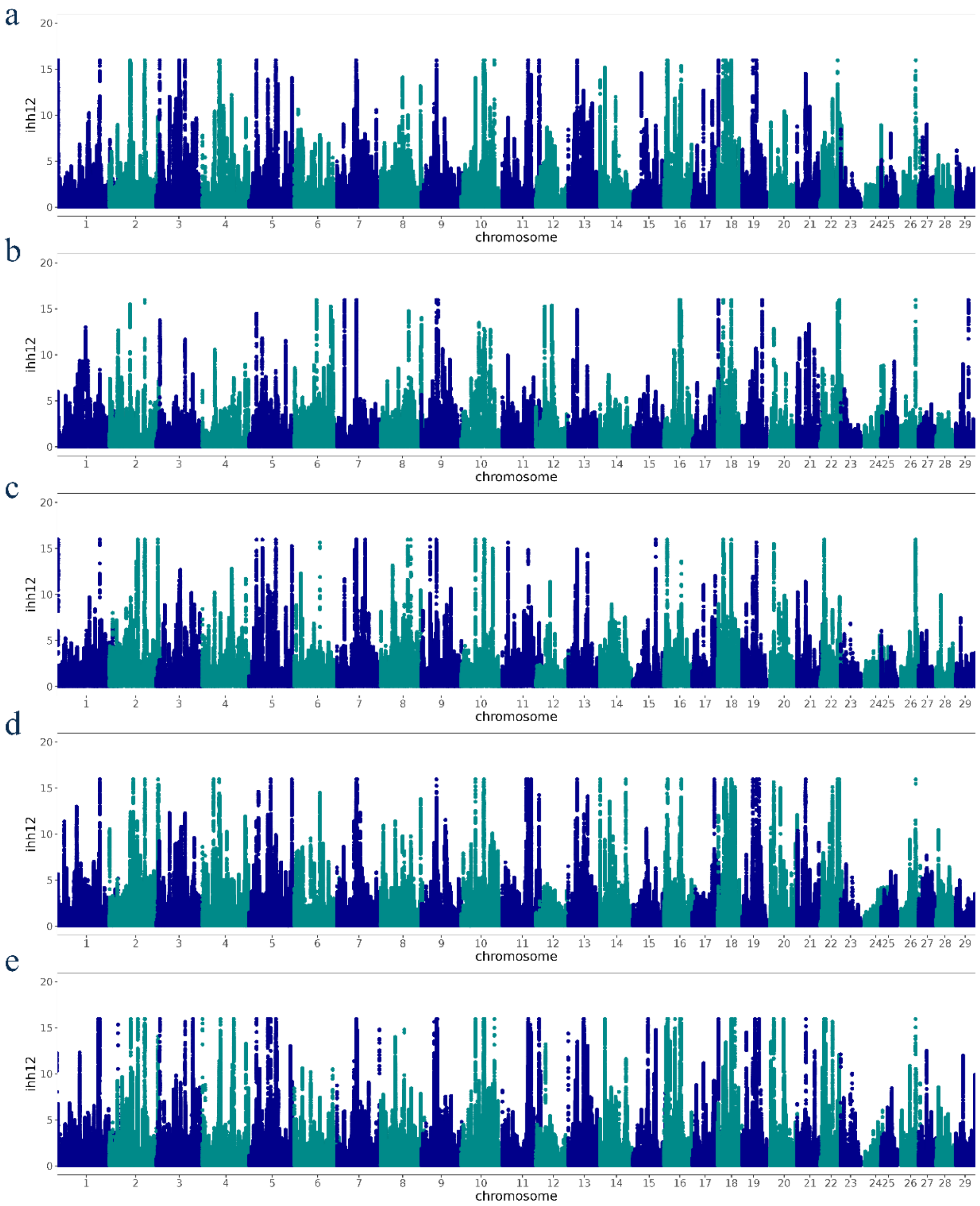

Supplementary Figure S8 Manhattan plots of ihh12 statistics in Hubei indigenous cattle breeds.

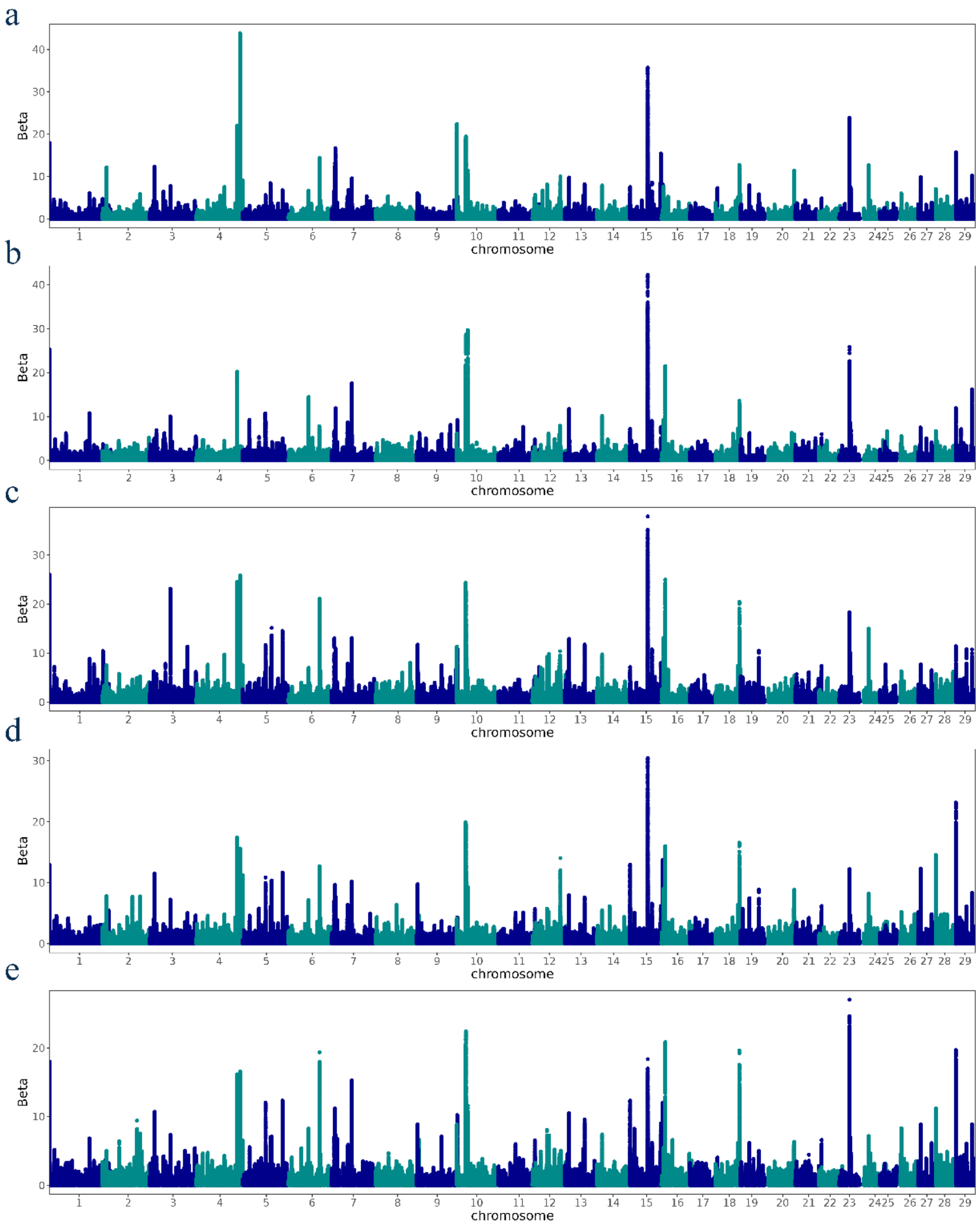

Supplementary Figure S9 Manhattan plots of Beta statistics in Hubei indigenous cattle breeds.

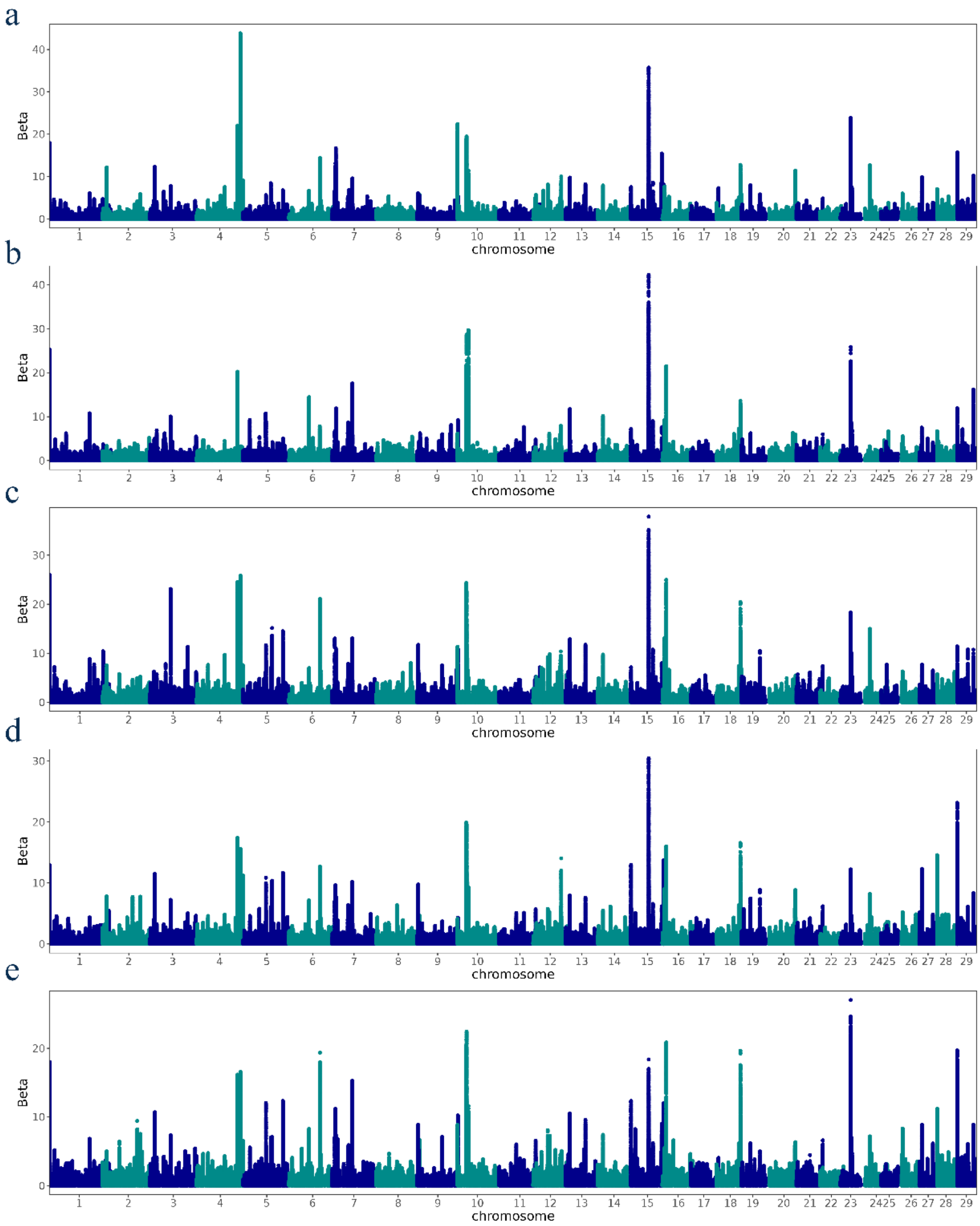

Supplementary Figure S10 Manhattan plots of Tajima's D statistics in Hubei indigenous cattle breeds.

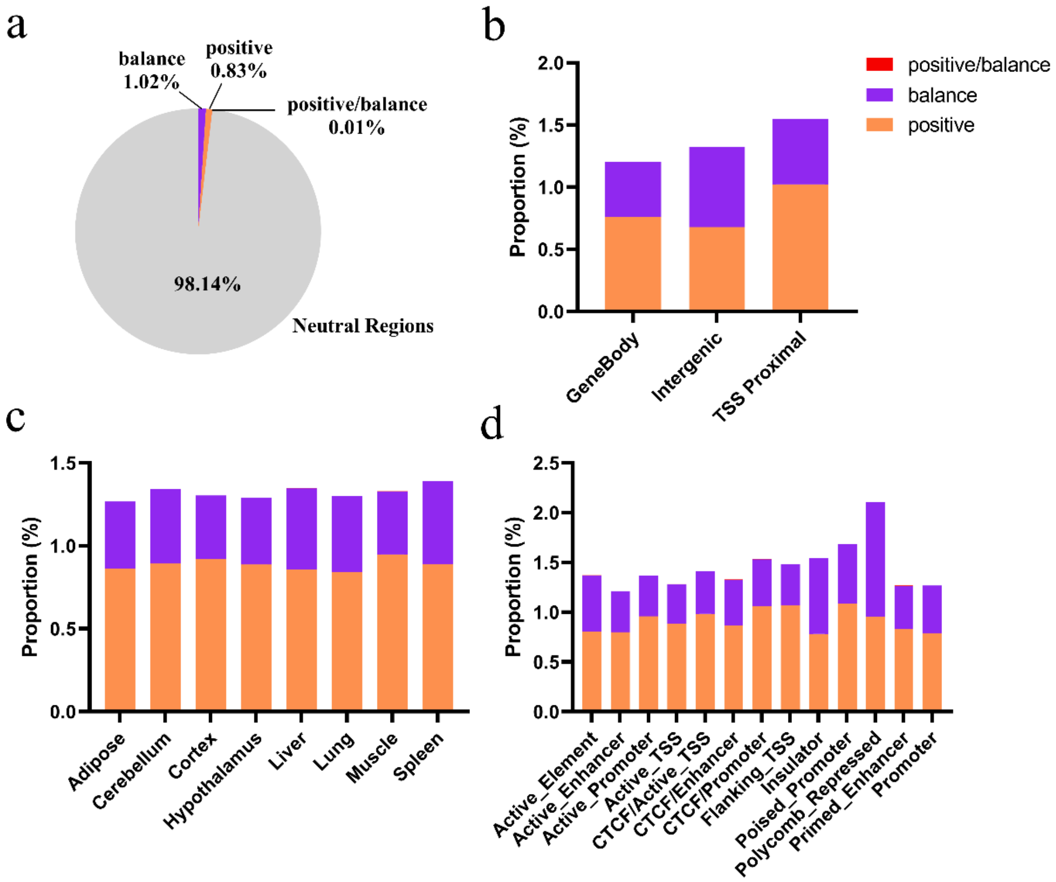

Supplementary Figure S11. Distribution of selection signals across genomic regions and cis-regulatory elements (CREs) in Dabieshan cattle. (a) Proportion of genomic regions under positive selection, balancing selection, and both across breeds. (b) Distribution of selection signals across gene body, intergenic regions, and TSS-proximal regions. (c) Tissue-specific distribution of adaptive selection signals. (d) Selection signal coverage across different CRE types. The Y-axis represents the proportion calculated as the total length of adaptive selection regions divided by the total length of each functional category.

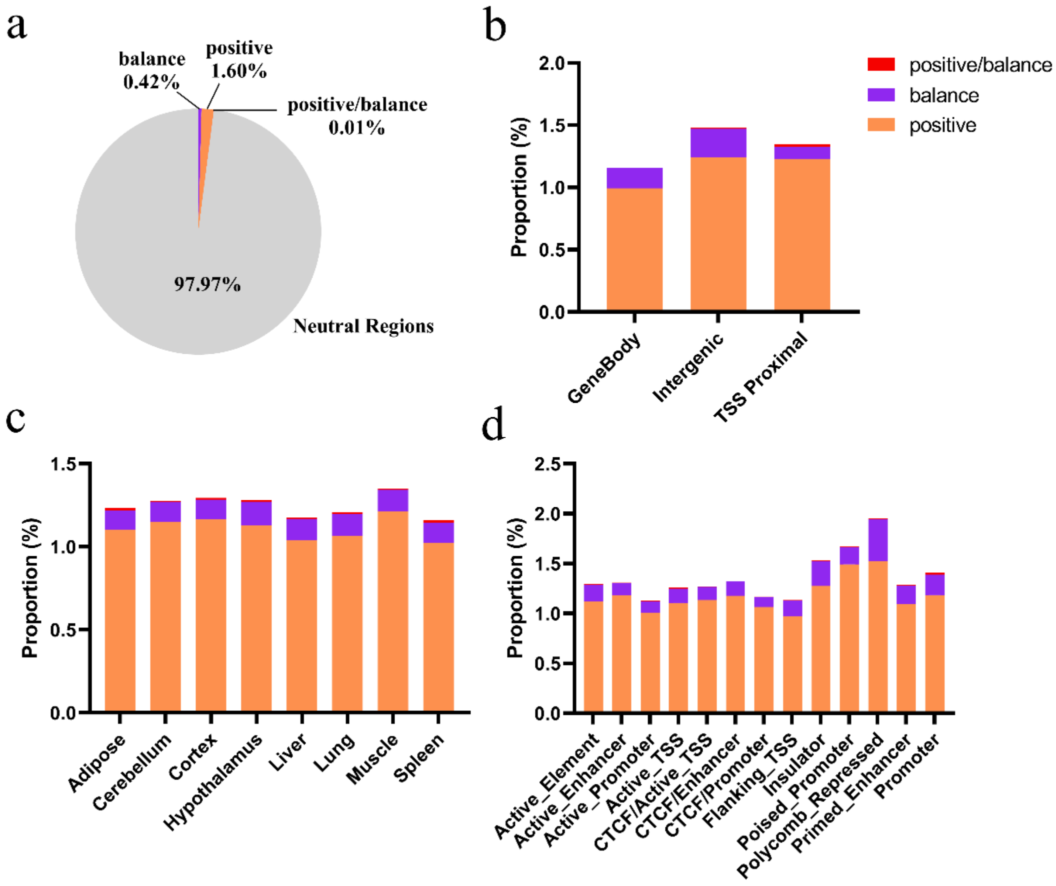

Supplementary Figure S12. Distribution of selection signals across genomic regions and cis-regulatory elements (CREs) in Wuling cattle. (a) Proportion of genomic regions under positive selection, balancing selection, and both across breeds. (b) Distribution of selection signals across gene body, intergenic regions, and TSS-proximal regions. (c) Tissue-specific distribution of adaptive selection signals. (d) Selection signal coverage across different CRE types. The Y-axis represents the proportion calculated as the total length of adaptive selection regions divided by the total length of each functional category.

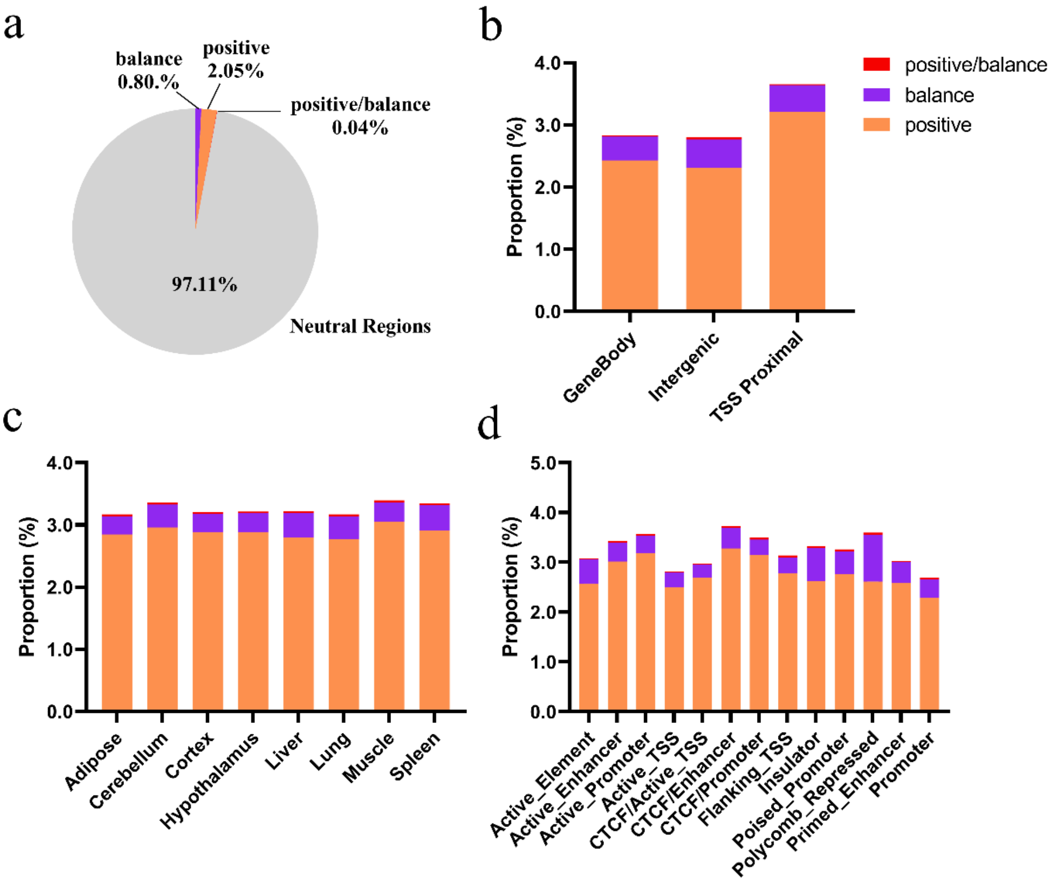

Supplementary Figure S13. Distribution of selection signals across genomic regions and cis-regulatory elements (CREs) in Yunba cattle. (a) Proportion of genomic regions under positive selection, balancing selection, and both across breeds. (b) Distribution of selection signals across gene body, intergenic regions, and TSS-proximal regions. (c) Tissue-specific distribution of adaptive selection signals. (d) Selection signal coverage across different CRE types. The Y-axis represents the proportion calculated as the total length of adaptive selection regions divided by the total length of each functional category.

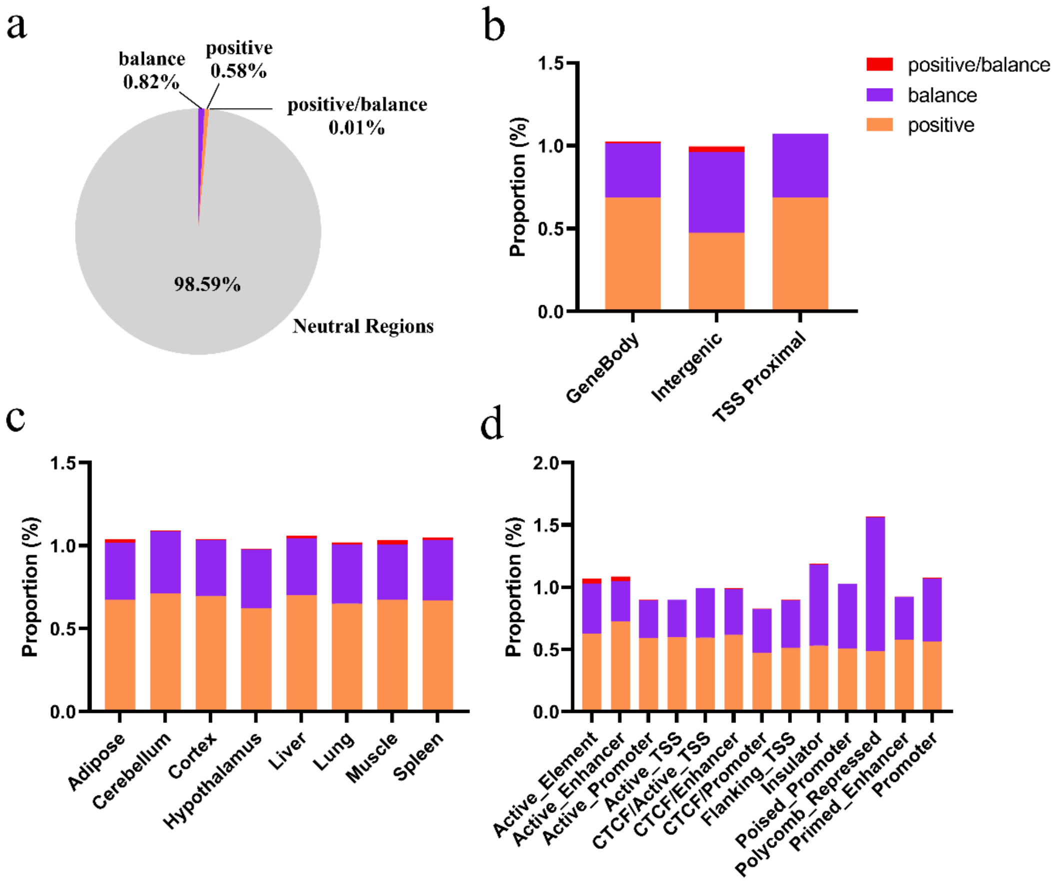

Supplementary Figure S14. Distribution of selection signals across genomic regions and cis-regulatory elements (CREs) in Zaobei cattle. (a) Proportion of genomic regions under positive selection, balancing selection, and both across breeds. (b) Distribution of selection signals across gene body, intergenic regions, and TSS-proximal regions. (c) Tissue-specific distribution of adaptive selection signals. (d) Selection signal coverage across different CRE types. The Y-axis represents the proportion calculated as the total length of adaptive selection regions divided by the total length of each functional category.

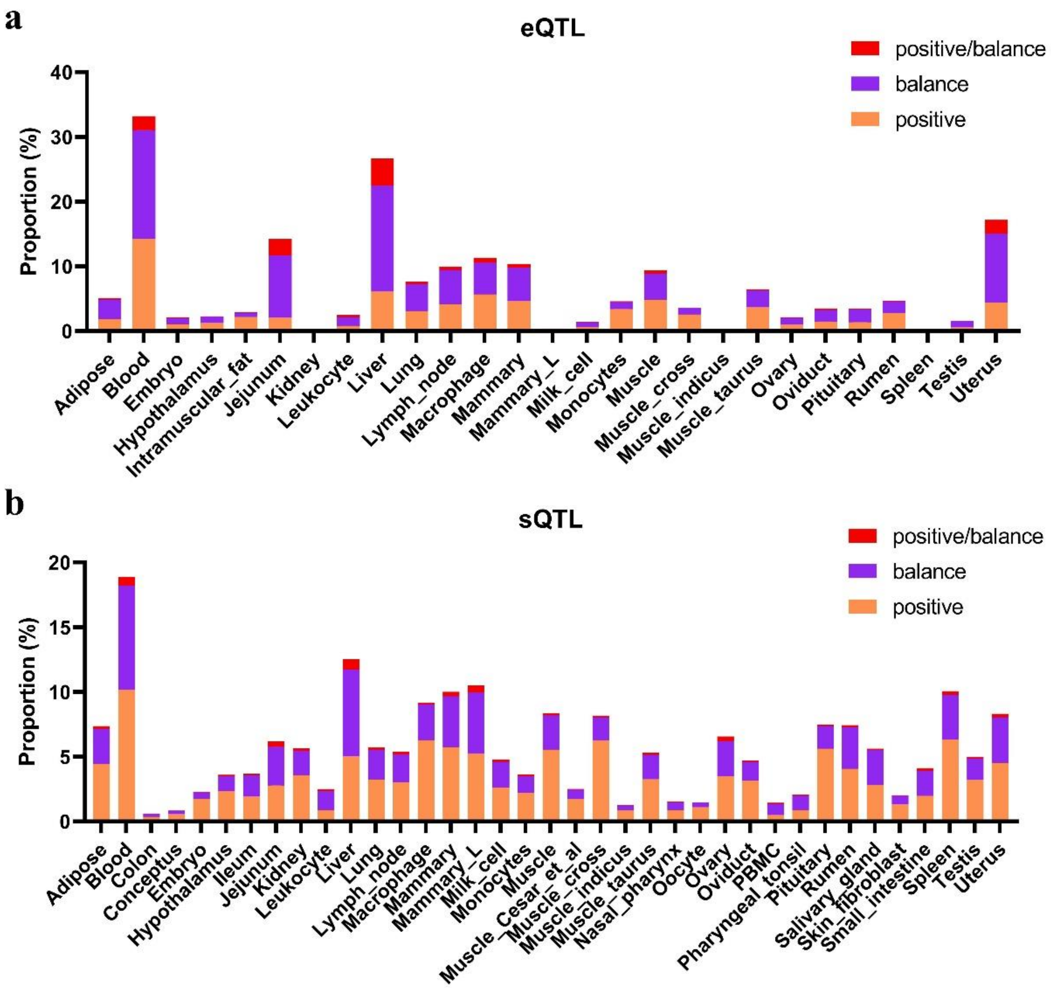

Supplementary Figure S15. Proportion of expression quantitative trait loci (eQTL) and splicing quantitative trait loci (sQTL) across different tissues in Dabieshan cattle. (a) Distribution of eQTL proportions in various tissues. (b) Distribution of sQTL proportions in the same tissues. The bars represent the proportion of positive (orange), balance (purple), and positive/balance (red) QTL effects in each tissue. The Y-axis represents the proportion calculated as the total length of adaptive selection regions divided by the total length of each functional category.

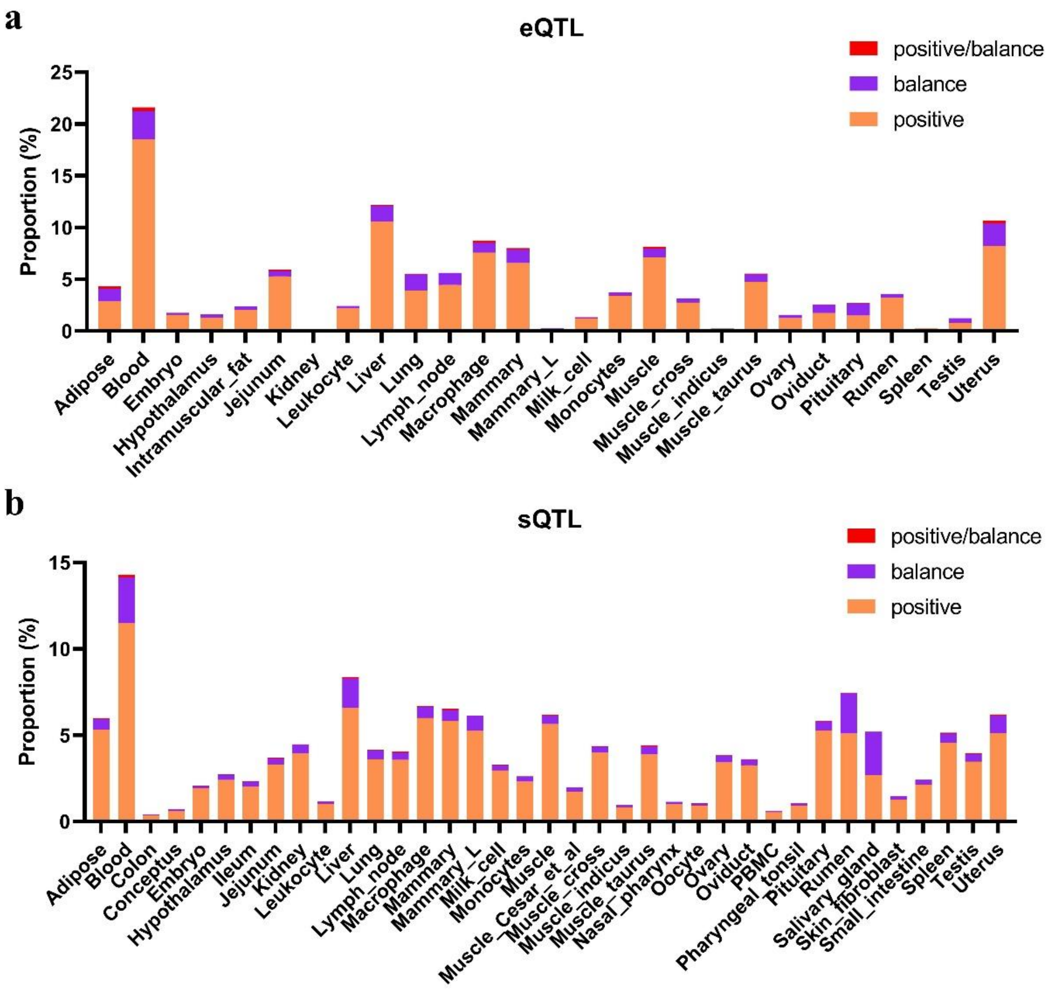

Supplementary Figure S16. Proportion of expression quantitative trait loci (eQTL) and splicing quantitative trait loci (sQTL) across different tissues in Wuling cattle. (a) Distribution of eQTL proportions in various tissues. (b) Distribution of sQTL proportions in the same tissues. The bars represent the proportion of positive (orange), balance (purple), and positive/balance (red) QTL effects in each tissue. The Y-axis represents the proportion calculated as the total length of adaptive selection regions divided by the total length of each functional category.

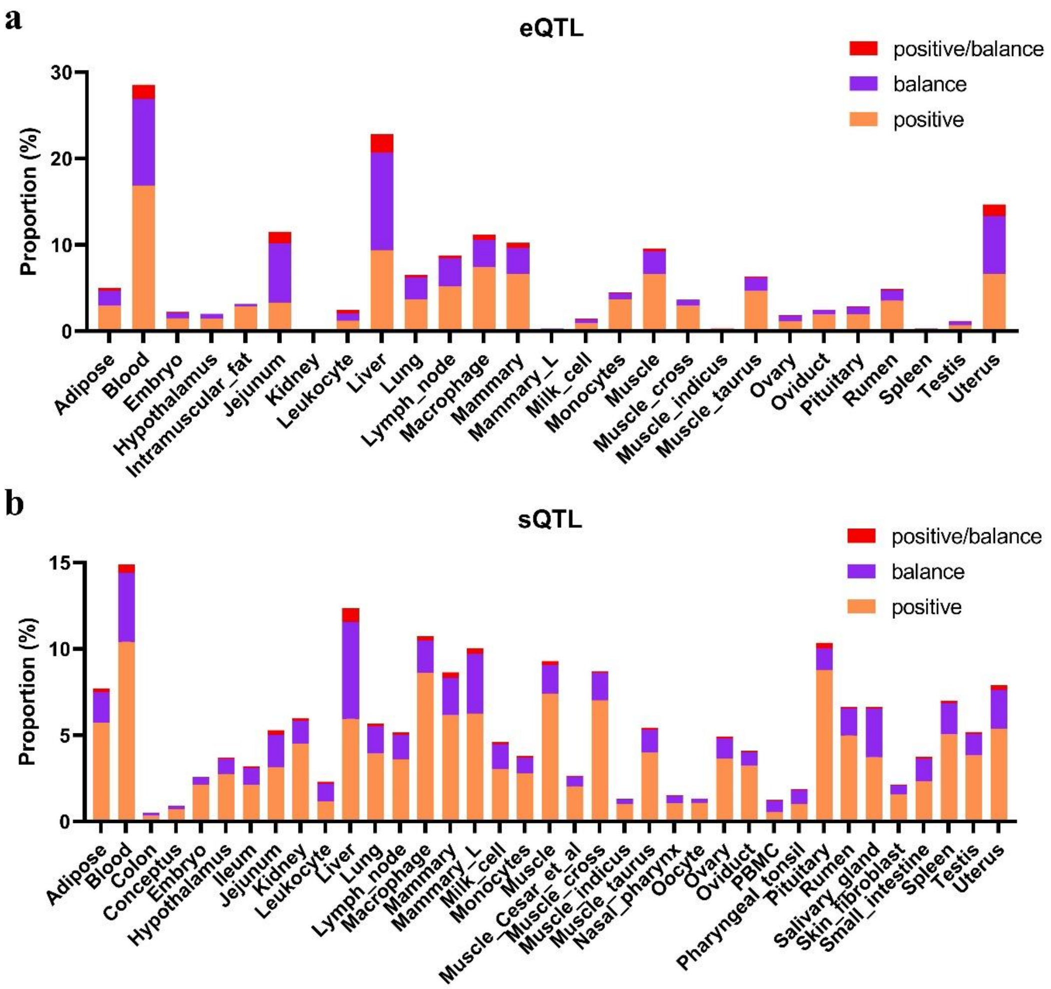

Supplementary Figure S17. Proportion of expression quantitative trait loci (eQTL) and splicing quantitative trait loci (sQTL) across different tissues in Yunba cattle. (a) Distribution of eQTL proportions in various tissues. (b) Distribution of sQTL proportions in the same tissues. The bars represent the proportion of positive (orange), balance (purple), and positive/balance (red) QTL effects in each tissue. The Y-axis represents the proportion calculated as the total length of adaptive selection regions divided by the total length of each functional category.

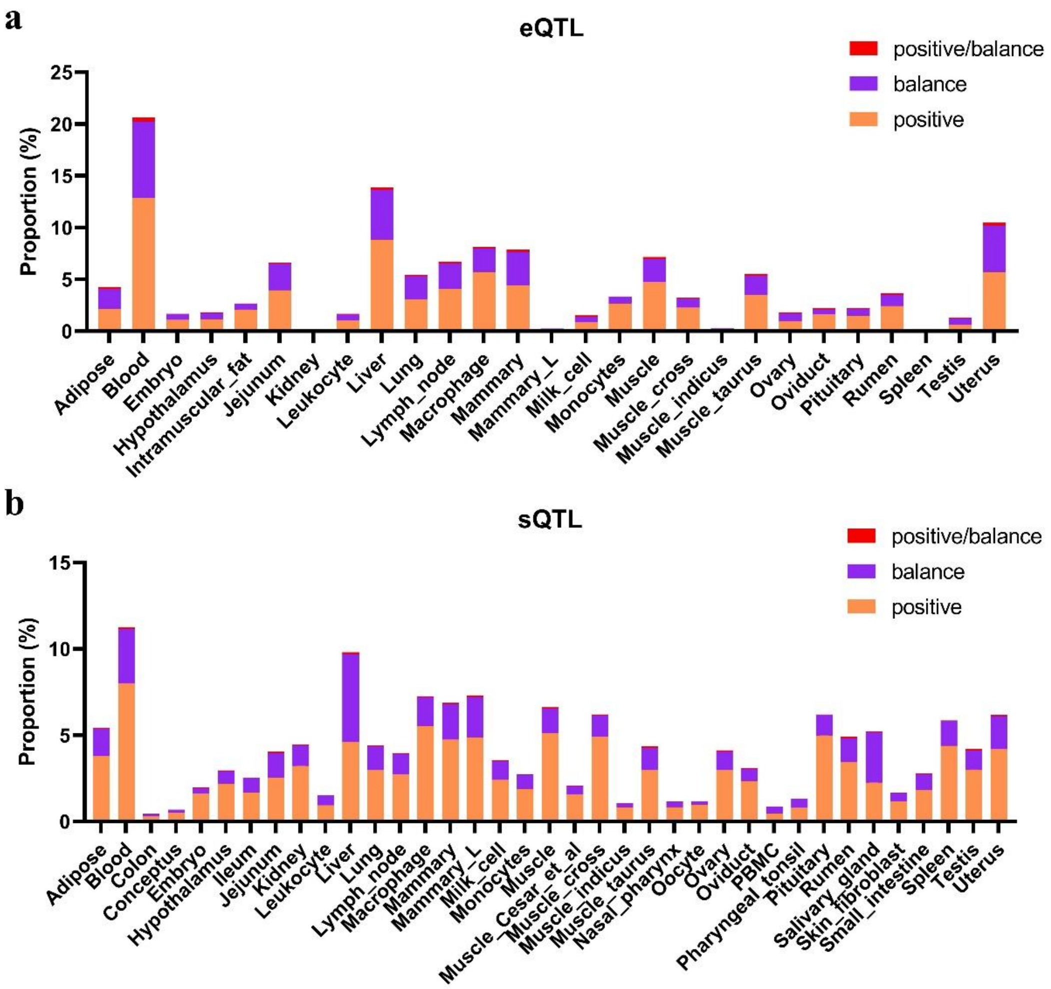

Supplementary Figure S18. Proportion of expression quantitative trait loci (eQTL) and splicing quantitative trait loci (sQTL) across different tissues in Zaobei cattle. (a) Distribution of eQTL proportions in various tissues. (b) Distribution of sQTL proportions in the same tissues. The bars represent the proportion of positive (orange), balance (purple), and positive/balance (red) QTL effects in each tissue. The Y-axis represents the proportion calculated as the total length of adaptive selection regions divided by the total length of each functional category.

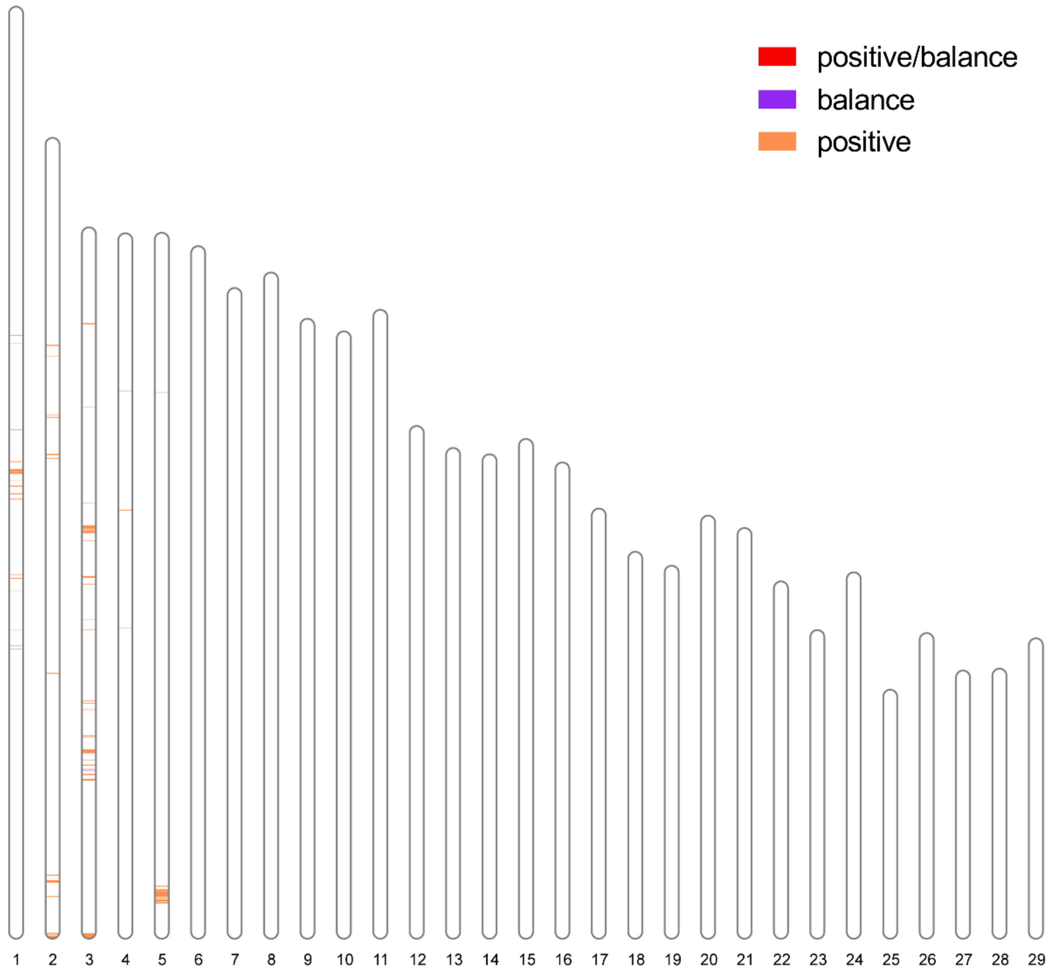

Supplementary Figure S19. Distribution of Candidate region in Positively Selected CREs in Dabieshan cattle.

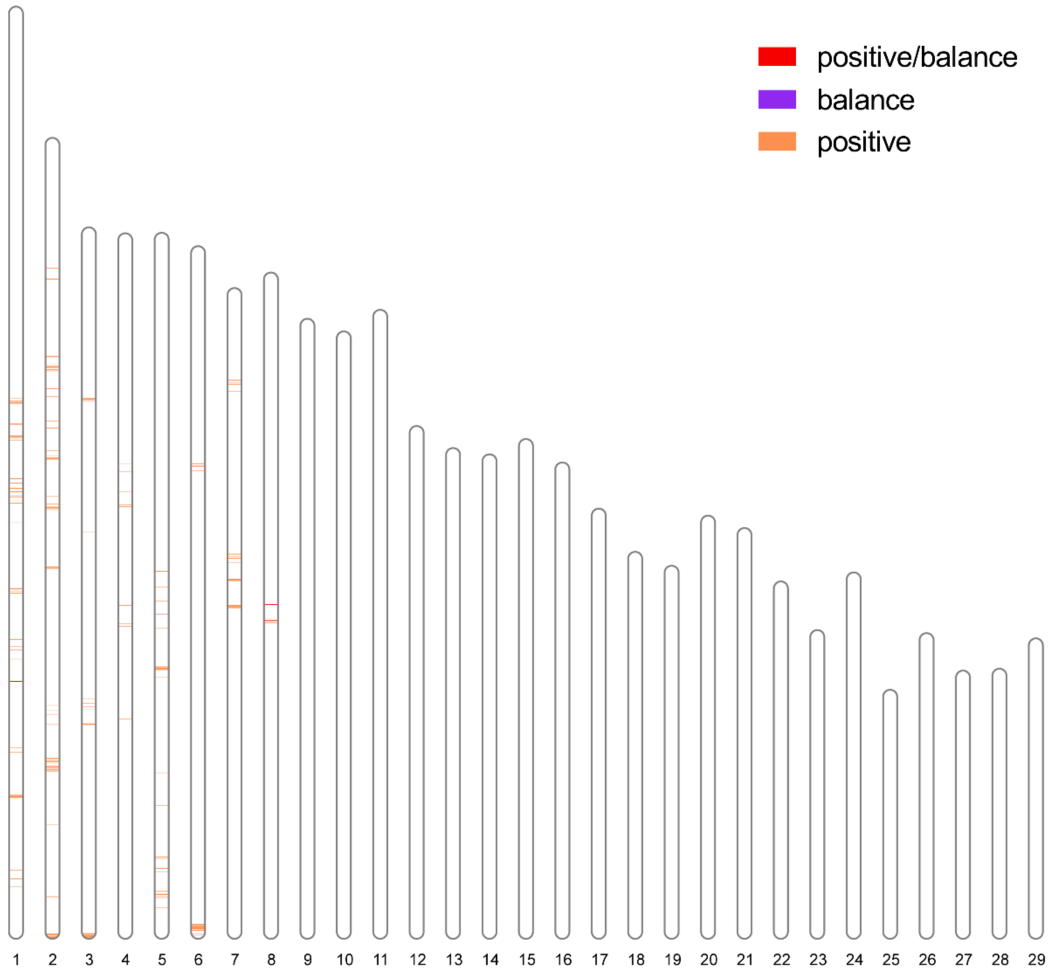

Supplementary Figure S20. Distribution of Candidate region in Positively Selected CREs in Wuling cattle.

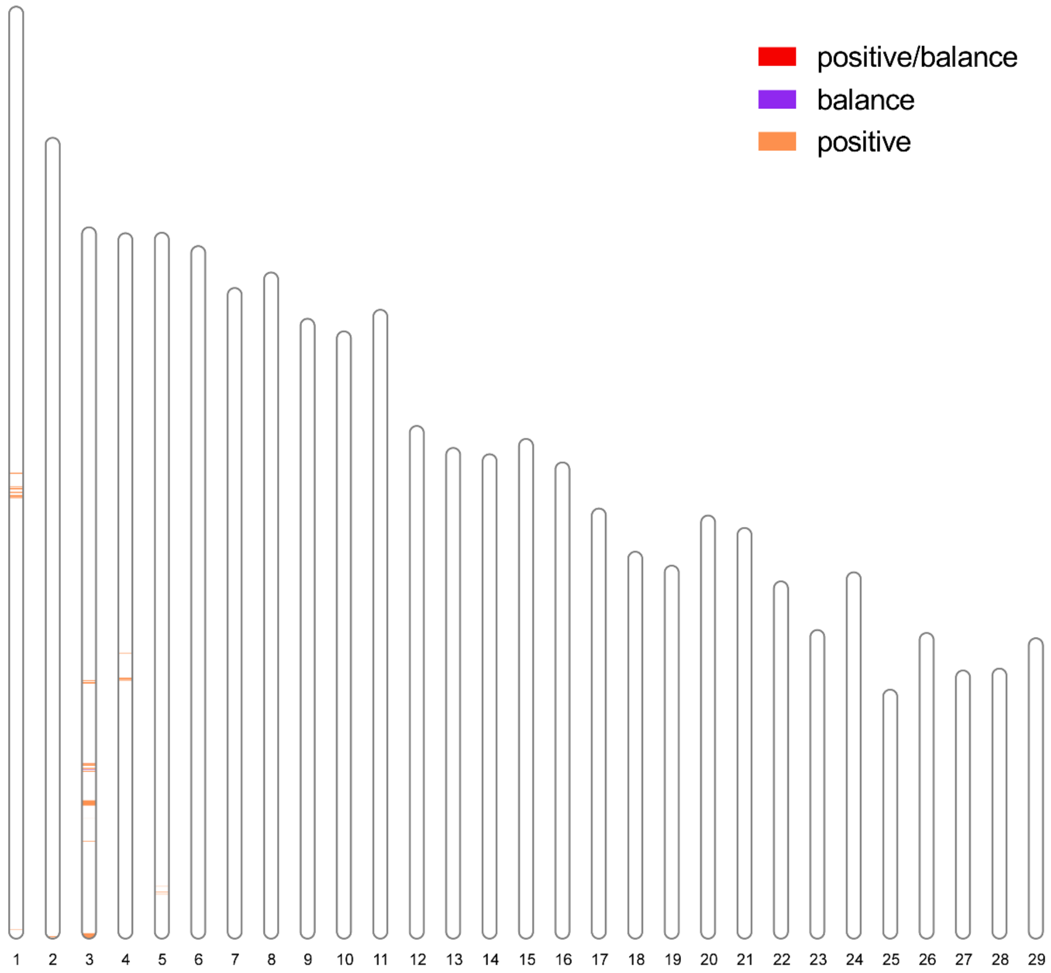

Supplementary Figure S21. Distribution of Candidate region in Positively Selected CREs in Yunba cattle.

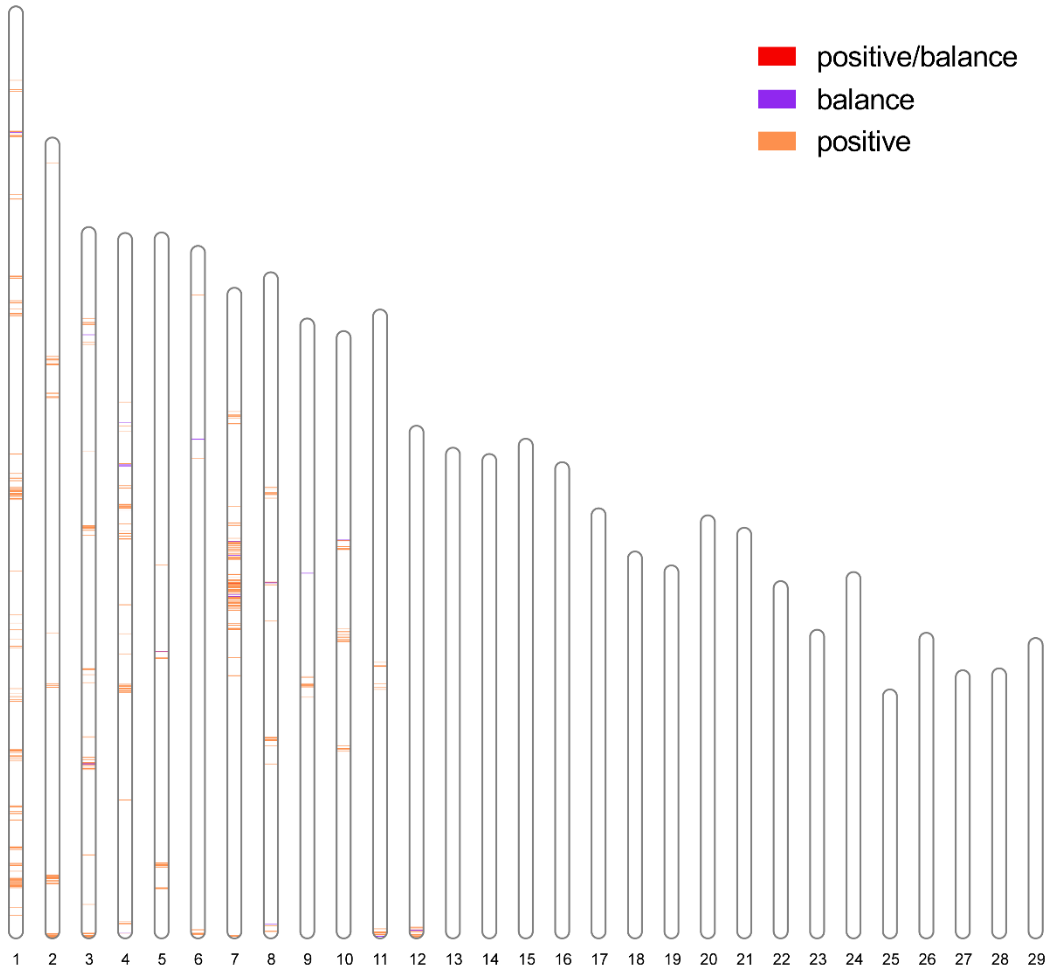

Supplementary Figure S22. Distribution of Candidate region in Positively Selected CREs in Zaobei cattle.
